# Supplementary material for: Nucleosome positioning sequence patterns as packing or regulatory
Source: PLoS Comput Biol. 2020 Jan 27;16(1):e1007365. doi: 10.1371/journal.pcbi.1007365 (PMC7004410; doi:10.1371/journal.pcbi.1007365)
Supplement: S1 Appendix — (PDF) [file pcbi.1007365.s001.pdf]

Patterns in nucleosomal DNA sequences: tools and examples.
Supplementary Appendix S1 to ‘Patterns in nucleosomal DNA as packing or regulatory‘.

Contents

1 Background **3**

2 Workflow **3**

2.1 Distribution of frequency of occurrence of dinucleotides along nucleosome sequences . . . 5

2.2 Cleavage site . . . 5

2.3 Determination of nucleosome position using dyad-symmetry of dinucleotide frequency profiles . . . 6

2.4 Patterns of dinucleotide frequency distributions and their periodograms 7

3 Dinucleotide shuffling **12**

4 Software implementation of dnpatterntools v1.0 **12**

4.1 Interface to Galaxy . . . 12

5 Figures **13**

List of Figures

A Example of transformation preceding a computation of a positional frequency profile of dinucleotide occurrences in all nucleosome sequences. The fasta sequence is transformed into a binary sequence of zeros and ones, in which ones indicate a presence of dinucleotide in that position. In this example the dinucleotide is AA. First column is a binary representation, second column is original sequence and third column shows a number of dinucleotide occurrences in the sequence. 14

B Gaussian smoothed coverage profile with window=70 on positive (+) and negative (-) strands. Identified peak maximums are shown with the red lines. 14

C A. Frequency profiles of selected dinucleotides on forward and complementary sequences of nucleosomes in mouse. Cleavage site is visibly expressed in profiles of all cell types. B. Pearson correlation coefficient between the profiles on forward and reverse complement sequences computed within sliding window of 146bp at the beginning positions of dinucleotide frequency profiles in A. 15

D AA/TT patterns and periodograms in human and mouse. 16

E CC/GG patterns and periodograms in human and mouse. 17

F WW/SS patterns and periodograms in human and mouse. 18

G WW pattern and periodogram in human and mouse. 19

H SS pattern and periodogram in human and mouse. 20

I RR/YY patterns and periodograms in human and mouse. 21

J Example of dinucleotide frequency profiles and their correlations in shuffled nucleosome sequences. Panel A. Frequency profiles computed from a batch of full length (219bp) mouse nucleosome sequences shuffled by *ushuffle* tool. Panel B. Correlation profile. 22

List of Tables

A Variability of 10bp vicinity period in periodograms of dinucleotide frequency distribution patterns in human CD4+ and apoptotic and control mouse NAC cells. 9
B Ranking of the patterns by 10bp spectral power in each condition. . . 11
C List of utilities in dnpatterntools 13

1 Background

The software tools and protocols to determine nucleosome occupancy and positioning from the micrococcal nuclease sequencing (MNnase-seq) data are established already [8, 36]. A nucleosome's occupancy and positioning information are obtained from profiles of peaks resulting from the MNase-seq data alignment to a reference genome of an organism under investigation. Peak positions usually are determined using a traditional gaussian [22] or improved wavelet smoothing [24]. In addition to positioning information (a) shift and occupancy change events for nucleosomes can be computed if data for cases and controls is available [8].

However, practical software tools to compute patterns of dinucleotide frequency distributions from nucleosome sequences have not been sufficiently described. We attempted to describe in more detail an algorithm used in computation of previously characterized patterns in nucleosomal DNA [12, 14]. We describe dnpatterntools v1.0 software utilities to reproduce patterns already characterized and to compute new patterns.

2 Workflow

A workflow to compute patterns of dinucleotide frequency distributions from nucleosome sequences consists of several steps:

1. computation of distribution of frequency of dinucleotide occurrences in a bulk of aligned sequences;

2. determination of nucleosome's position in the sequences;

3. selection and symmetrization of dinucleotide frequency profiles from the determined interval;

4. computation of frequency profiles of composite dinucleotides WW/SS (W = A or T and S=C or G) and RR/YY (R=A or G and Y=C or T) ;

5. application of normalization and smoothing to the frequency profiles to remove noise and to compute periodograms.

To illustrate this workflow practically we use three datasets of nucleosome sequences: from human CD4+ cells [25], from apoptotic lymphocyte cells [4] and from nucleus accumbens cells of mouse brain [30].

The sequences from the CD4+ and apoptotic lymphocyte cells were characterized previously [12]. The nucleosome sequences from mouse nucleus accumbens brain cells [30] were obtained from NCBI GEO archive under accession GSE54263. The Fastq files with the accessions SRR1138261, SRR1138262, SRR1138263 representing MNase-seq 99bp length reads of three biological replicates from control mice were aligned to *mm9* reference genome by BWA [17]. The BAM files were converted into BED and genome coverage was computed by BEDTools [23]. The profile of peaks from genome coverage files was generated by Gaussian smoothing using 70bp window size [32]. Genome-wide peak summits exceeding value of 10 were discarded. An example of smoothed coverage signal (shaded areas) and summits (red lines) is presented in Figure B. The summits, positions of which were closest to gene transcription start sites were selected. The reads overlapping these summits were extended by 20bp in 5' direction and 100 bp in 3' direction [7] resulting in the 219 bp long sequence (99 bp read plus 120 bp extensions). The DNA sequences in these regions were extracted from mouse mm9 reference by BEDTools, aligned by the experimental 5'-end and formatted into Fasta file.

2.1 Distribution of frequency of occurrence of dinucleotides along nucleosome sequences

In the given nucleosome sequences at each position an occurrence of dinucleotide is coded by 1. All other dinucleotides (positions) are 0 as illustrated by Figure A. Frequency of occurrence of dinucleotide at a single position along the sequence is simply a sum of occurrences of that dinucleotide normalized by the number of sequences. The *diprofile*program (see Table C computes frequency distribution of a single dinucleotide along sequences in Fasta file. The `dnp-subset-dinuc-profile.sh` script calls *diprofile* and computes frequency distribution of all dinucleotides along sequences in Fasta file in a forward original and in a complementary representation of each sequence. Patterns of dinucleotide frequency distributions represent statistical sequence-specific features of nucleosomal DNA. Patterns originating from different organisms, conditions or experimental manipulations may have signatures characteristic only to that particular condition [14].

2.2 Cleavage site

Nucleosomal DNAs are generally obtained from the purified chromatin stabilized with formaldehyde and digested with MNnase which cleaves sequence specific linker sites [24]. However, sequence fragments resulting from MNase digestion have substantial variability from 10 to 20bp in the precise fragment ends [9]. In dinucleotide frequency profiles computed from nucleosome sequences obtained by MNase-seq and aligned by experimental end a cleavage site manifests as a narrow large peak because of the sequence specificity. In both, profiles of dinucleotide frequencies of mouse cells and of human CD4+ and apoptotic lymphocyte cells the region of a cleavage site can be identified by a large peak at the beginning of the computed frequency profiles. A cleavage site peak is a positive indicator that the batch of sequences obtained from MNase-seq data contains sequences of nucleosomes. Panel A of Figure C shows the large peak at a cleavage site region present in the beginning of the frequency profiles in all datasets - mouse and human.

2.3 Determination of nucleosome position using dyad-symmetry of dinucleotide frequency profiles

Dyad-symmetry is a hallmark of the nucleosome DNA sequence [20]. The peak arrangements in patterns of dinucleotide (most often AA, TT , AT, CC, GC or GG) frequency distribution along the nucleosomal sequence have a recognizable dyad-symmetry. These dinucleotides statistically are preferred [11] and are periodically distributed along nucleosome DNA sequence [20]. These dinucleotide preferences were investigated and reported by studies in vitro [19],[2], statistically [6, 9], from analysis of nucleosome stability [11] and computationally [3, 5, 14]. Dyad-symmetry feature helps to determine a position of a nucleosome in a bulk of sequences aligned by experimental end - because at the nucleosome position centered on the dyad the forward and complementary profiles of dinucleotides will have a maximum positive correlation. Panel B in Figure C shows Pearson correlation coefficient at each position along the sequence computed between forward (fw) and reversed complement (rc) of frequency profiles for selected dinucleotides within the window corresponding to the nucleosome size of 146bp. In such obtained matrix of Pearson correlation coefficients a maximum positive correlation between of fw and rc frequency profiles of either AA, TT, TA, CC, GG and GC dinucleotides or combination will indicate a nucleosome position - same for all sequences in a batch.

A challenge is that identification of nucleosome position via correlation cannot be fully automated, because correlations vary in both: along the sequence and in different conditions. The position in which fw and rc frequency profiles of one or several dinucleotides attain maximum positive correlation has to be at a close proximity to the cleavage site which is indicator of a nucleosome start. In mouse strongest correlations were found between fw and rc frequency profiles for AA/TT dinucleotides. However, in human cells the strongest correlations were found for AT and GC dinucleotides. Panel B of Figure C shows correlation profiles obtained for all three cases: nucleosomes in mice brain, human CD4+ cells and apoptotic lymphocyte cells. Solid dark red line represents inferred a most likely start position of

the nucleosome from the dinucleotide frequency profiles. For mouse it is at a position 25 from the beginning of the sequences aligned by the experimental end; for human this position is 105 and for apoptotic lymphocyte cells it is 109. The correlation profiles for apoptotic lymphocyte cells is a good illustration that the cleavage site is to be used as a guide, because strong positive correlations are observed also in regions in which a presence of nucleosome is unlikely.

2.4 Patterns of dinucleotide frequency distributions and their periodograms

Nucleosome sequences in yeast are characterized by a very clear pattern of AA/TT frequency distribution with peaks occur each 10 base pairs [14, 29]. In other organisms dinucleotides other than AA may elicit clearer patterns of frequency distributions. For example, in human, mouse and flies the GC/CG/CC/GG dinucleotide periodicity correlates better with nucleosome positioning [18, 25, 33]. It was also shown that composite patterns of RR/YY (purine-purine / pyrimidine-pyrimidine) can be associated with nucleosome stability [14]. In addition to frequency distribution of all 16 dinucleotides we separately compute distribution of frequencies of composite dinucleotides strong-weak SS/WW (S = C or G, W= A or T) and purine/pyrimidine RR/YY (R = A or G, Y = C or T). This is done by the `dnp-compute-composite.sh` utility (Table C).

The frequency distributions on original forward sequences and their complement should be equally represented in the pattern. Therefore, for each dinucleotide its fw and rc patterns are averaged at each position. This step is called symmetrization. The symmetrized patterns of dinucleotide frequency distributions are computed by utility `dnp-symmetrize.sh`. Finally, to improve a representation of the patterns they are smoothed by applying a moving average filter. Usually, a several positions of a pattern are trimmed from both ends, since most often they represent noise. Smoothing is accomplished with the program *Fourier_Transform* that is wrapped into `dnp-smooth.sh` utility, which also applies trimming. Our experience suggests

that the best size for moving average filter is 3 positions and optimal trimming is 4 positions from each end.

A spectral decomposition of a pattern reveals the strongest periodical components that make the pattern. The dinucleotide frequency distributions in nucleosome sequences are expected to have peaks at 10bp and periods multiple of 10. Spectral decomposition may serve as means to identify a leading dinucleotide pattern in each condition. Table A summarizes periods of peaks around 10bp across conditions and major patterns in the data used in this workflow, illustrating variability in peak periods around 10base pairs. by performing random sampling with replacement. From nucleosome fasta sequences of apoptotic lymphocytes (n=711873), CD4+ cells (n=58150750) and control mice NAC (n=210000) the 50% of sequences 31 times were randomly sampled by seqtk sample function. For each sample of the sequences the dnpatterntools pattern computation pipeline was applied and pattern periodograms of the dinucleotides and conditions were computed. In the [9.5, 11] bp interval of the periodogram a period of a maximal peak was identified. From a sample of 31 period of maximal peak a mean period and its standard error were estimated. These estimates are shown in Table A. ANOVA analysis of the sampled (10bp vicinity) period means in different dinucleotides and conditions did not reveal significant differences between the dinucleotides or conditions. Scripts and results of this estimation procedure can be found on dnpatterntools github site https://github.com/erinijapranckeviciene/dnpatterntools under periods.

In Section 5 the patterns and periodograms are shown for the dinucleotides of interest AA/TT, CC/GG, WW/SS and RR/YY in Figures D,E,F and I. Because of the substantial differences in scale, the WW and SS plots are also presented separately in Figures G and H. The AA/TT, CC/GG, WW/SS and RR/YY patterns of human CD4+ and apoptotic lymphocyte cells reported in [12] are identical to the patterns reproduced in this study. This result ensures that patterns from the nucleus accumbens brain cells of control mice [30] can be compared with the patterns previously obtained, since they were computed by the same pipeline, which generated a reproducible result.

Dinucleotide Pattern	Mean period of peak closest to 10bp ± its standard error Apoptotic	CD4+	Mouse
AA	10.225 ± 0.095	10.061 ± 0.078	10.293 ± 0.078
TT	10.077 ± 0.092	10.216 ± 0.077	10.216 ± 0.084
CC	10.341 ± 0.079	10.138 ± 0.089	10.177 ± 0.087
GG	10.132 ± 0.084	10.229 ± 0.081	10.045 ± 0.081
SS	10.283 ± 0.082	9.983 ± 0.073	10.154 ± 0.077
WW	10.151 ± 0.091	10.193 ± 0.086	10.267 ± 0.092
RR	10.203 ± 0.092	10.232 ± 0.077	10.216 ± 0.074
YY	10.329 ± 0.093	10.364 ± 0.0845	10.203 ± 0.087

Table A: Variability of 10bp vicinity period in periodograms of dinucleotide frequency distribution patterns in human CD4+ and apoptotic and control mouse NAC cells.

The plotting commands and data are available on github in the same repository as dnpatterntools.

To identify patterns that have a most pronounced spectral power at 10bp range we used above described sampling data. The 10bp period integral spectral power was calculated by summing all components from [10.1 bp, 10.9 bp] interval of the periodogram. The dnpatterntools fourier function computes periodogram that is detrended and normalized which makes integral spectral power comparable across different periodograms. Samples of integral spectral power at 10bp period were computed for the patterns of each AA, TT, CC, GG, SS, WW, RR and YY dinucleotide in each apoptotic lymphocytes, human CD4+ cells and control mouse NAC 31 times from 50% of sequences each time sampled from the original fasta (apoptotic lymphocytes (n=711873), CD4+ cells (n=58150750) and control mice NAC (n=210000)) using seqtk sample with replacement. For each of these dinucleotides and conditions 31 instances of integral spectral power at 10bp period was computed.

The means of the spectral power between the dinucleotides in each condition were compared using ANOVA test implemented in R. To estimate which differences are significant in multiple hypothesis comparison we used TukeyHSD function im-

plemented in R. Table B shows ranking of the patterns by a magnitude of the mean of the integral spectral power at 10bp period, its standard deviation and standard error for each dinucleotide and condition. The patterns for which the spectral power at 10bp period was significantly different from majority of other patterns (p.adjusted < 0.05) in multiple comparisons are indicated by * in Table B. Results of multiple hypothesis comparison and plots produced by TukeyHSD function supporting Table B are available on dnpatterntools github site under *periods*.

Dinucleotide	Mean spectral power at 10bp period ± sd (stderr)
Apoptotic lymphocytes	
RR^*	$0.1037 \pm 0.0286(0.0051)$
YY^*	$0.1010 \pm 0.0254(0.0046)$
WW	$0.0992 \pm 0.0258(0.0046)$
SS	$0.0975 \pm 0.0311(0.0056)$
CC	$0.0810 \pm 0.0210(0.0038)$
GG	$0.0808 \pm 0.0222(0.0040)$
AA	$0.0805 \pm 0.0181(0.0033)$
TT	$0.0761 \pm 0.0174(0.0031)$
CD4+ cells	
RR^*	$0.1113 \pm 0.0303(0.0054)$
YY^*	$0.1096 \pm 0.0264(0.0047)$
SS^*	$0.0988 \pm 0.0344(0.0062)$
WW	$0.0915 \pm 0.0219(0.0039)$
GG	$0.0767 \pm 0.0179(0.0032)$
TT	$0.0765 \pm 0.0195(0.0035)$
AA	$0.0758 \pm 0.0191(0.0034)$
CC	$0.0735 \pm 0.0190(0.0034)$
Control mouse nucleus accumbens cells	
YY^*	$0.1082 \pm 0.0276(0.0050)$
RR^*	$0.1036 \pm 0.0293(0.0053)$
SS	$0.0992 \pm 0.0309(0.0055)$
WW	$0.0989 \pm 0.0338(0.0061)$
AA	$0.0812 \pm 0.0232(0.0042)$
TT	$0.0805 \pm 0.0216(0.0039)$
CC	$0.0776 \pm 0.0147(0.0026)$
GG	$0.0775 \pm 0.0166(0.0030)$

Table B: Ranking of the patterns by 10bp spectral power in each condition.

3 Dinucleotide shuffling

Random shuffling of nucleotides in sequences is a technique that creates randomness and destroys a periodical structure of dinucleotide occurrences in nucleosome sequences. To make our tools complete we included as extra tool a wrapper to the uShuffle program [16] in the utility `shuffle_sequences.sh`. Figure J illustrates application of shuffling to mouse nucleosome sequences. Panel A shows dinucleotide frequency distributions in shuffled nucleosome sequences of mouse. Panel B depicts correlation profiles computed from shuffled profiles. Obviously, randomized sequences do not have structure that was observed in original sequences (see panel A in Figure C). Similarly, the correlation profiles do not have any prominently expressed correlation value that could be associated with the likely nucleosome position compared to the real data as in panel B in Figure C.

4 Software implementation of dnpatterntools v1.0

The dnpatterntools consist of core programs and tool utilities. The core programs are written in C++ using a SeqAn library [10]. The SeqAn library is a collection of C++ header definitions of functions specifically written to work with genomic data (Fasta, BAM and VCF). A C++ program that uses SeqAn library depends only on the SeqAn function headers. The tool utilities in dnpatterntools are written in shell as commands that integrate also into Galaxy environment [1]. Each has a corresponding fully functional Galaxy xml wrapper. Table C summarizes core programs and tool utilities. Input and output and functionality of each utility is demonstrated by a test script which is a part of the package. The dnpatterntools are available in github repository `https://github.com/erinijapranckeviciene/dnpatterntools`.

4.1 Interface to Galaxy

All tool utilities in dnpatterntools have fully functional interface to Galaxy [1]. A Planemo configured Galaxy server can be launched calling *planemo serve* from within the tools directory on a local Linux machine. The Galaxy workflow of dnpatterntools

Core utilities	
C++	Description
binstrings	Binary strings from sequences, more in Figure A.
diprofile	Distribution of dinucleotide frequency in sequences.
corrprofile	Correlation between forward and reverse complement profiles.
Fourier_Transform	Computes periodogram, normalization and smoothing [12].
**Tool utilities**	
Shell	Description
`dnp-binary-strings.sh`	Binary strings for multiple dinucleotides.
`dnp-compute-composite.sh`	Composite WW/SS and RR/YY dinucleotide profiles.
`dnp-correlation-between_profiles.sh`	Correlations for all dinucleotides.
`dnp-fourier-transform.sh`	Periodogram for all dinucleotides.
`plot-selected.sh`	Gnuplot of selected columns.
`dnp-select-range.sh`	Select profiles within interval.
`dnp-smooth.sh`	Smoothing by moving average.
`dnp-subset-dinuc_profile.sh`	Frequency profiles of all dinucleotides.
`dnp-symmetrize.sh`	Symmetrization of frequency profiles.

Table C: List of utilities in dnpatterntools

utilities is described in the document galaxy-workflow.pdf in the dnpatterntools github repository.

5 Figures

```
00000001000001100100000000000000000000000000000000000000000000000000000000000000000000000000000000000000000001000000000000000000000001
10000011000000000000100000000000000000001000001100000000000000000000000000000000000000000000000000000000000000000000000000000000000
00000000000000000000000000000000000000000000000001000000000000000000000000000000001100000000000001000000000000000000000000000001000
00100000000       AA              CAGCTGGAAGGCCAAAGAACCAGGGGCTCAGGTGAGAGAGGGCAGGGGCTGGCGGCCACAGCAGGGCCAGGCATCGCCAGACCCACCACCAGGGCC
CCATGTGGCCAATTTCTAGTCCCCTCTGTTCCCAAATCACAAAGCCATCCTCCAAGTTGTCCATCCCATGTCCAAGGTCAAAGGCAGAGCCCTTCCTGCTTCTCCTCACGGGCCCCTGGTGCCCACATAC
TGGCCTGGGTGACGAGGTCAGTCCAGCCACTCCACCTGCCCGGAGCCTCCAGCCCATCAGGCCTGAGGGGGCATGGCCTCCCCAACCTAGTGCAGCCTGGGGCTTCCCCTCCCTGGAAACGCCTGGTTCT
GGCCAGTTCTCCAACACCTACCCCCTCTCCAAGTCGAATCATCCG       19
01100100000000000000000000000000000000000000000000000000000000000000000000000000000000000000001000000000000000000000000110000011000
00000001000000000000000000010000011000000000000000000000000000000000000000000000000000000000000000000000000000000000000000000000000
00000000000000000000000000000000000001000000000000000000000000000000001100000000000000000000000010000000000000000010000010000000000
00000000000       AA              CAAAGAACCAGGGGCTCAGGTGAGAGAGGGCAGGGGCTGGCGGCCACAGCAGGGCCAGGCATCGCCAGACCCACCACCAGGGCCCCATGTGGCCAA
TTTCTAGTCCCCTCTGTTCCCAAATCACAAAGCCATCCTCCAAGTTGTCCATCCCATGTCCAAGGTCAAAGGCAGAGCCCTTCCTGCTTCTCCTCACGGGCCCCTGGTGCCCACATACTGGCCTGGGTGA
CGAGGTCAGTCCAGCCACTCCACCTGCCCGGAGCCTCCAGCCCATCAGGCCTGAGGGGGCATGGCCTCCCCAACCTAGTGCAGCCTGGGGCTTCCCCTCCCTGGAAACGCCTGGTTCTGGCCAGTTCTCC
AACACCTACCCCCTCTCCAAGTCGAATCATCCGGGCACGGCCCTG       18
00000000000000000010000000000000000000000110000011000000000001000000000000000000010000011000000000000000000000000000000000000000000
00000000000000000000000000000000000000000000000000000000000000000000000000000000000000000001000000000000000000000000000000000110000
00000000000000000000100000000000000000100000100000000000000000000000000000000000000000001100000000000000100000000000000000000011000
00001000000       AA              CCAGGGCCCCATGTGGCCAATTTCTAGTCCCCTCTGTTCCCAAATCACAAAGCCATCCTCCAAGTTGTCCATCCCATGTCCAAGGTCAAAGGCAGA
GCCCTTCCTGCTTCTCCTCACGGGCCCCTGGTGCCCACATACTGGCCTGGGTGACGAGGTCAGTCCAGCCACTCCACCTGCCCGGAGCCTCCAGCCCATCAGGCCTGAGGGGGCATGGCCTCCCCAACCT
AGTGCAGCCTGGGGCTTCCCCTCCCTGGAAACGCCTGGTTCTGGCCAGTTCTCCAACACCTACCCCCTCTCCAAGTCGAATCATCCGGGCACGGCCCTGGCCGCCTGGCACTGTTTCCAAACCCTCGCCC
TGGTCTCAAGTCATAGTGCGCTAGATCTGAAACCCAGGAAGTCAC       21
00000000000000000000000000000000110000000000000000000000001000000000000000000100000100000000000000000000000000000000000000001100000
00000000000100000000000000000000110000000100000010000000000000000000000000000000000000000000000000000000000000000000000000000000000
00000000000000000001000000000000000000000000000000000000000000000000000000000000000000000000000000000000000000000110000000000000000000
00000000000       AA              ACCTAGTGCAGCCTGGGGCTTCCCCTCCCTGGAAACGCCTGGTTCTGGCCAGTTCTCCAACACCTACCCCCTCTCCAAGTCGAATCATCCGGGCAC
GGCCCTGGCCGCCTGGCACTGTTTCCAAACCCTCGCCCTGGTCTCAAGTCATAGTGCGCTAGATCTGAAACCCAGGAAGTCACAACACACCCCCAGGTCCCCTCGCCGAGCCGCACCCGCTCTTTGCCAC
TGATCTCCAGCTGGATCTCCCGGTCACGCAGCTTGCGCCAGTGGCTGTAGTACAAGGTCAGGGGTGTCCCCTCTTCCCGGGTCAGCTTCTCCCAGGCTTCCTGGGGGGTTGGGGGAGTTCAGGGTCATGC
CTCACCCTGGGCAAACCCCCACATGTAGCTGGGGCTATACCCTGG       15
```

Figure A: Example of transformation preceding a computation of a positional frequency profile of dinucleotide occurrences in all nucleosome sequences. The fasta sequence is transformed into a binary sequence of zeros and ones, in which ones indicate a presence of dinucleotide in that position. In this example the dinucleotide is AA. First column is a binary representation, second column is original sequence and third column shows a number of dinucleotide occurrences in the sequence.

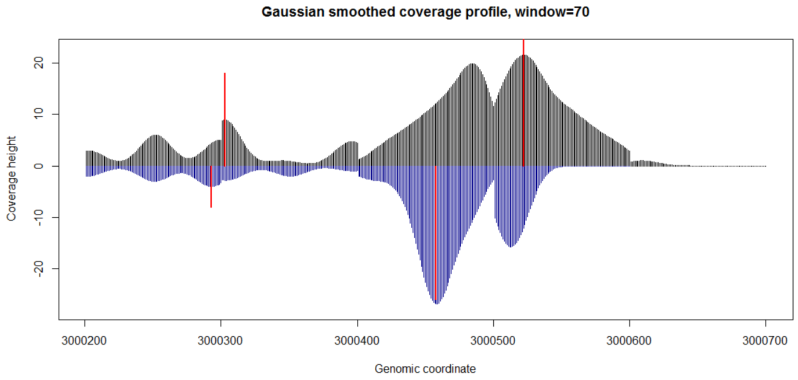

Figure B: Gaussian smoothed coverage profile with window=70 on positive (+) and negative (-) strands. Identified peak maximums are shown with the red lines.

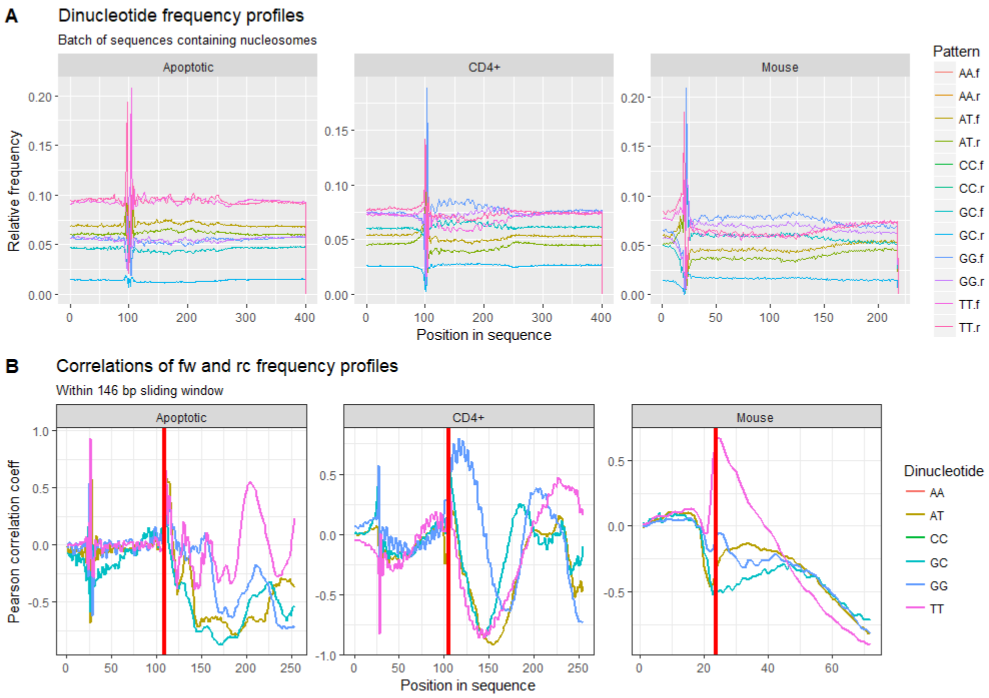

Figure C: A. Frequency profiles of selected dinucleotides on forward and complementary sequences of nucleosomes in mouse. Cleavage site is visibly expressed in profiles of all cell types. B. Pearson correlation coefficient between the profiles on forward and reverse complement sequences computed within sliding window of 146bp at the beginning positions of dinucleotide frequency profiles in A.

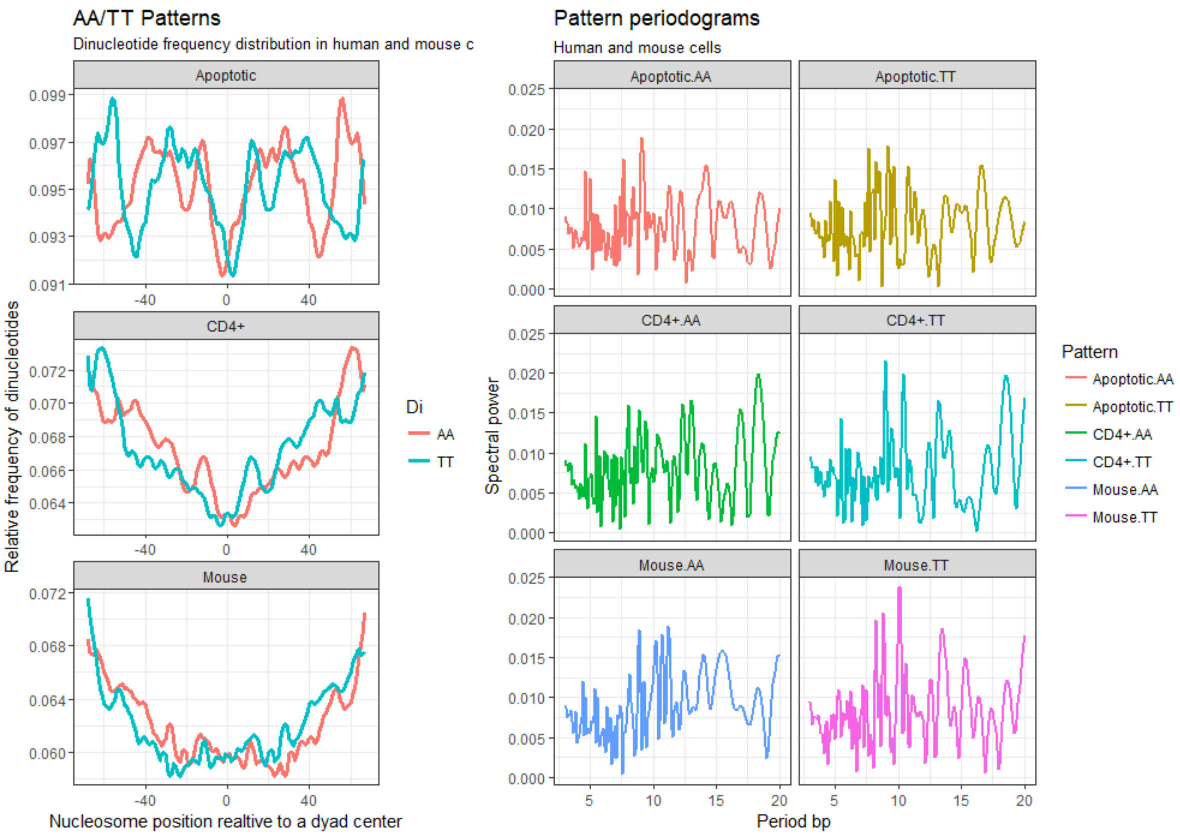

Figure D: AA/TT patterns and periodograms in human and mouse.

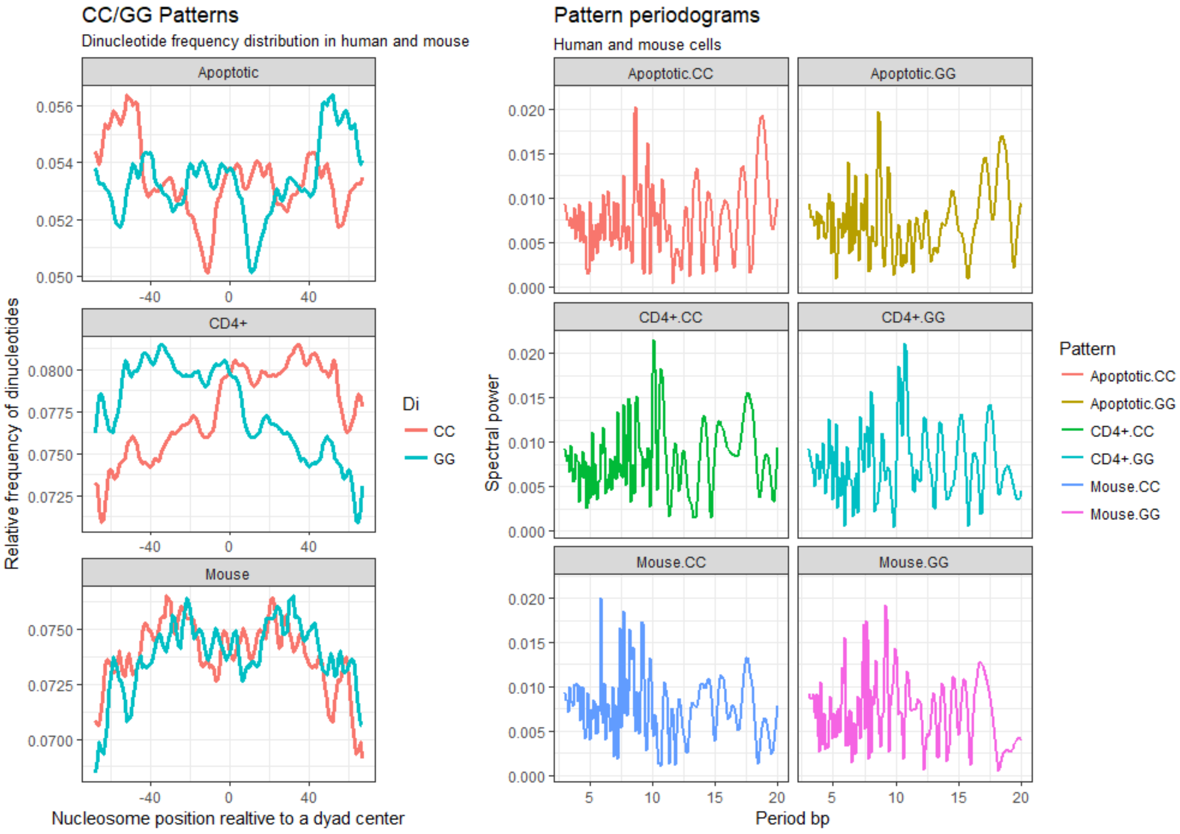

Figure E: CC/GG patterns and periodograms in human and mouse.

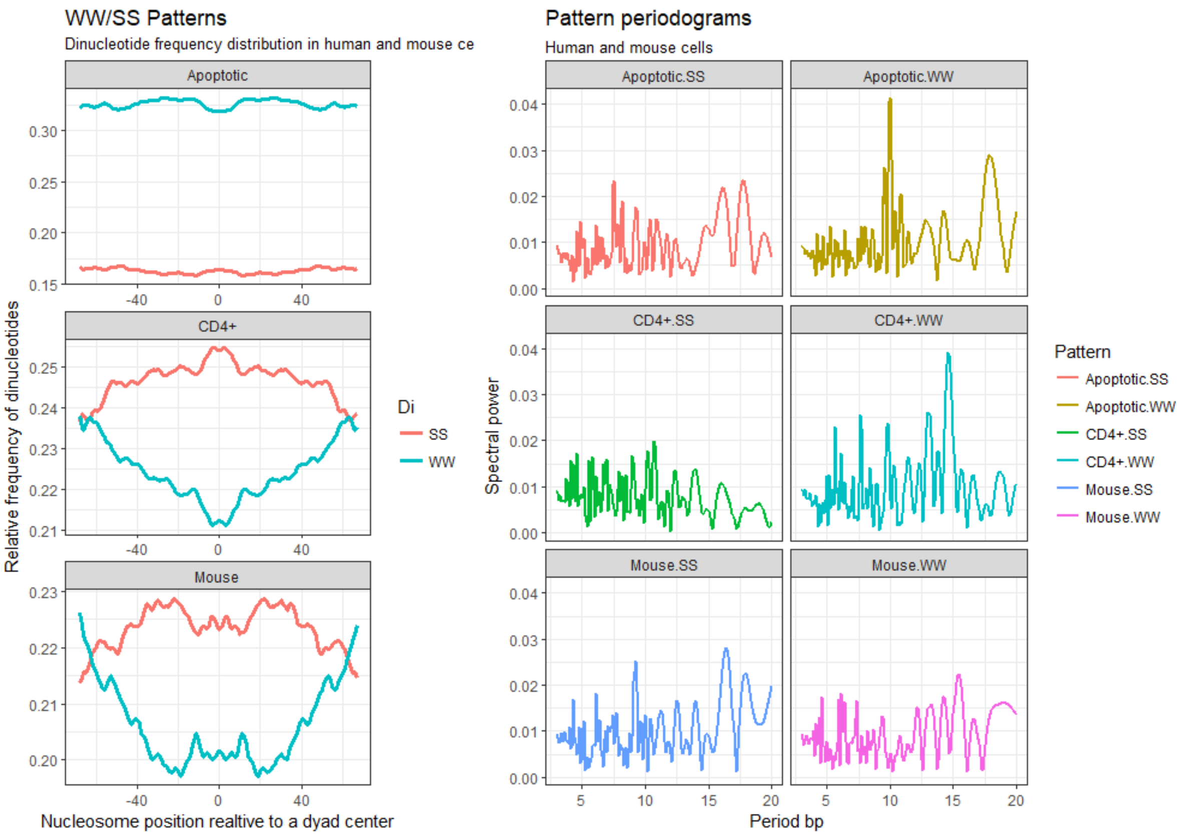

Figure F: WW/SS patterns and periodograms in human and mouse.

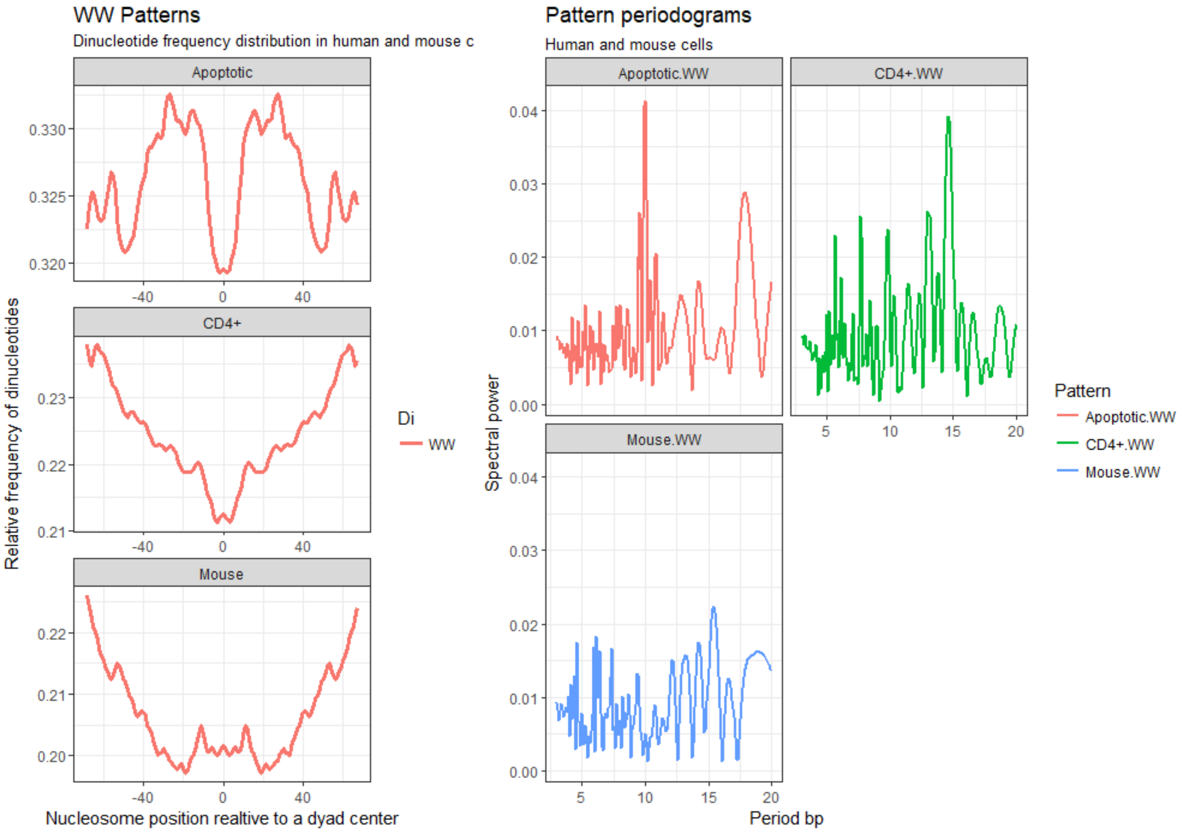

Figure G: WW pattern and periodogram in human and mouse.

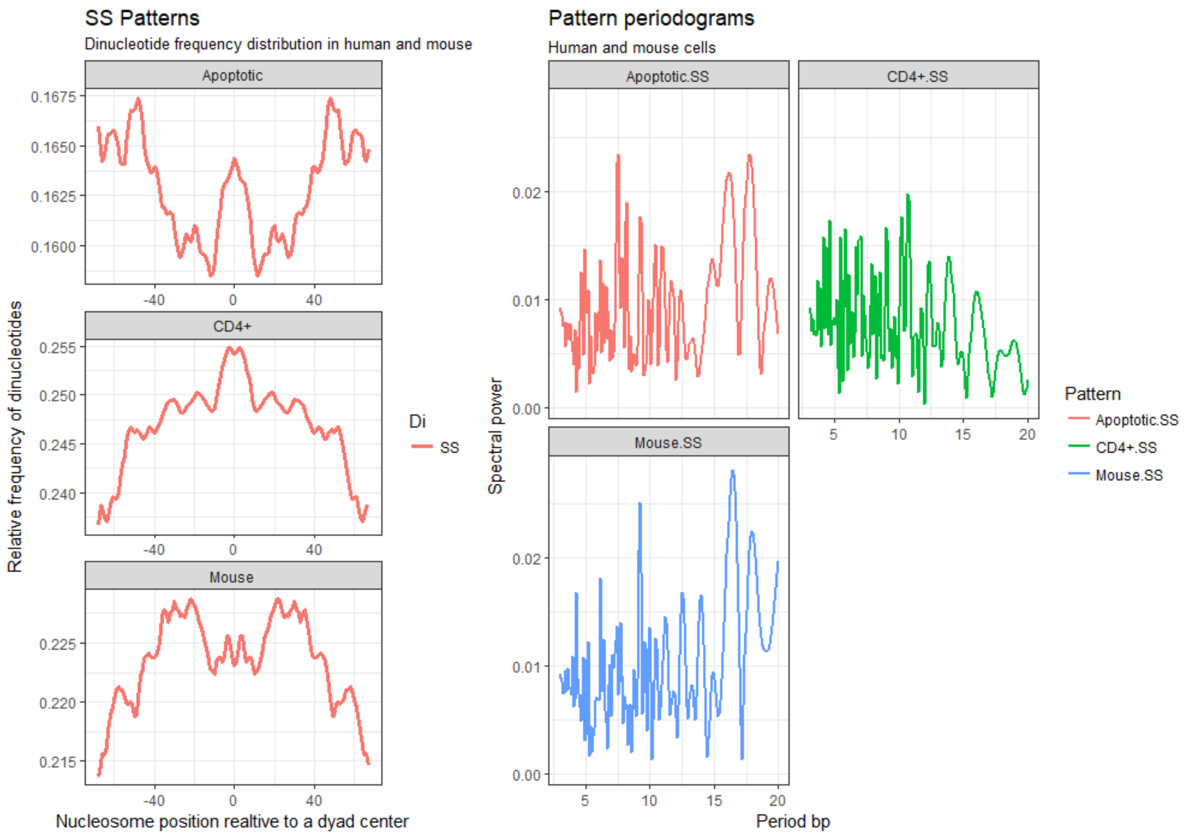

Figure H: SS pattern and periodogram in human and mouse.

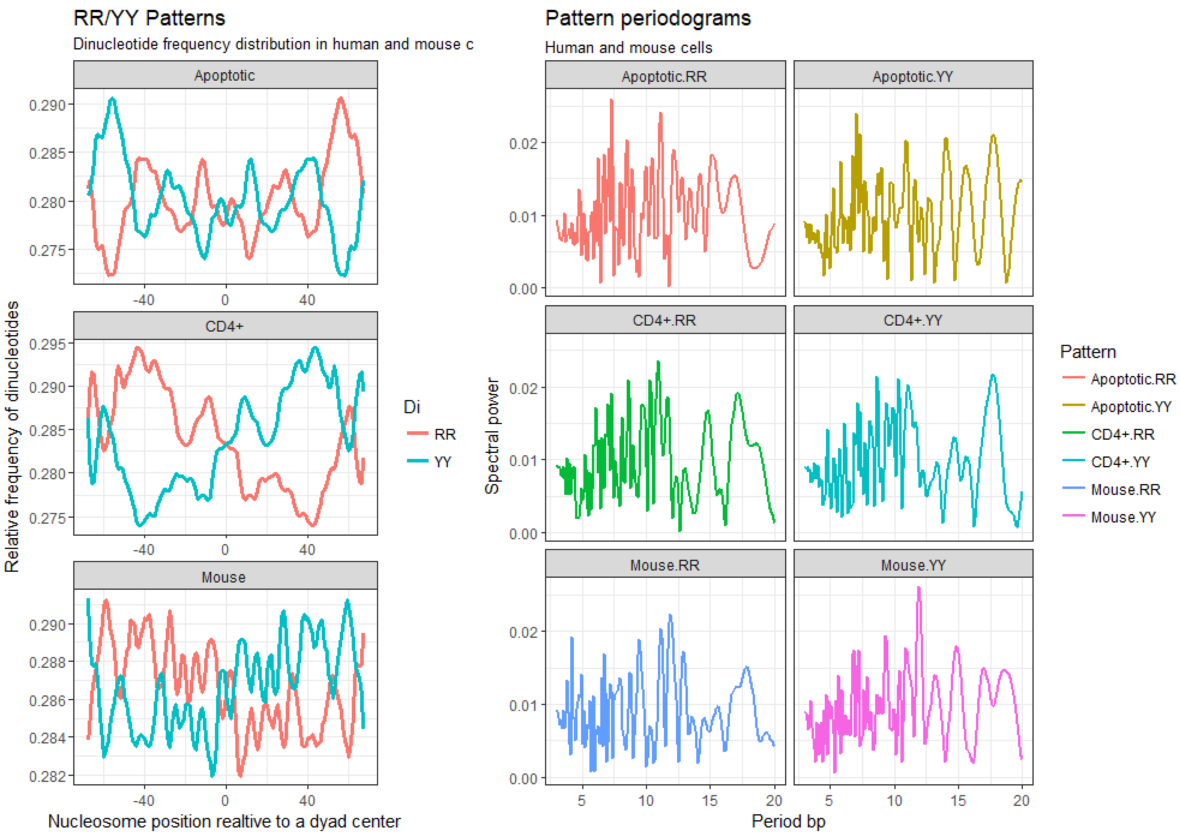

Figure I: RR/YY patterns and periodograms in human and mouse.

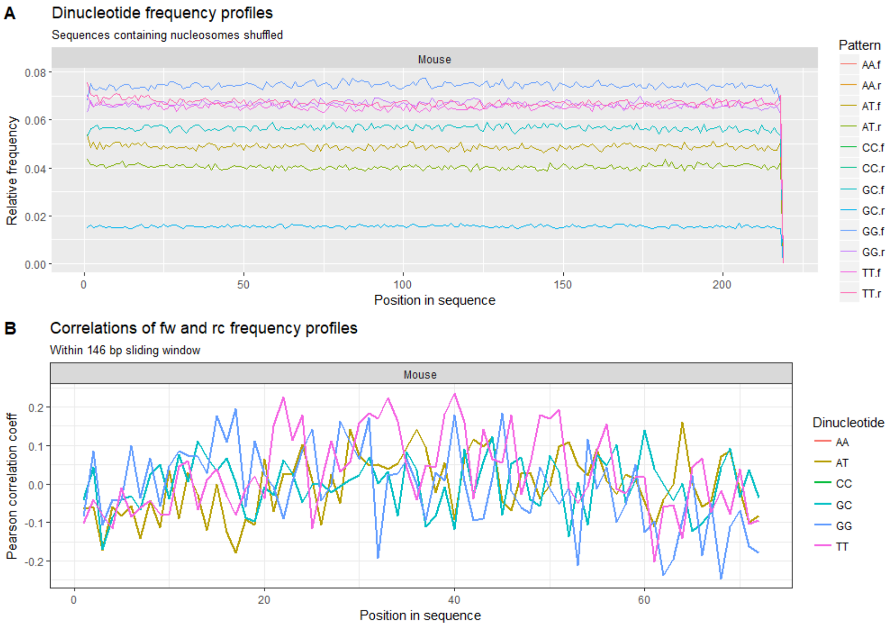

Figure J: Example of dinucleotide frequency profiles and their correlations in shuffled nucleosome sequences. Panel A. Frequency profiles computed from a batch of full length (219bp) mouse nucleosome sequences shuffled by *ushuffle* tool. Panel B. Correlation profile.

References

[1] Afgan, E. et al. (2018) The Galaxy platform for accessible, reproducible and collaborative biomedical analyses: 2018 update. Nucleic Acids Res. 46(W1), W537-W544.

[2] Beh, L.Y. et al. (2015) DNA-guided establishment of nucleosome patterns with coding regions of a eukaryotic genome. Genome Res. 25(11), 1727-1738.

[3] Bettecken, T. and Trifonov, E. N. (2009). Repertoires of the nucleosome positioning dinucleotides. PloSONE, 4(11), e7654.

[4] Bettecken, T. et al. (2012) Apoptotic cleavage of DNA in human lymphocyte chromatin shows high sequence specificity. J. Biomol. Struct. Dyn. , 30(2), 211-216.

[5] Bolshoy, A. (1995) CC dinucleotides contribute to the bending of DNA in chromatin. Nat. Struct. Biol., 2(6), 446-448.

[6] Bolshoy, A. et al. (1996) Applicability of the multiple alignment algorithm for detection of weak patterns: periodically distributed DNA pattern as a study case. Comput.Appl.Biosci.,12(5), 383–389.

[7] Brunelle, M. et al. (2017) Hgh-resolution genome-wide mapping of nucleosome positioning and occupancy level using paired-end sequencing technology. Methods Mol. Biol., 1528, 229-243.

[8] Chen, K. et al. (2013) DANPOS: dynamic analysis of nucleosome position and occupancy by sequencing. Genome Res., 23(2), 341-351.

[9] Chung, H. R. and Vingron, M. (2009) Sequence-dependent nucleosome positioning. J. Mol. Biol., 386(5), 1411-1422.

[10] Doring, A. et al. (2008) SeqAn an efficient generic C++ library for sequence analysis. BMC Bioinformatics, 9,11.

[11] Eslami-Mossalam, B. et al. (2016) Nucleosome dynamics: Sequence maters. Adv Colloid Interface Sci., 232, 101-113.

[12] Hosid, S. and Ioshikhes, I. (2014) Apoptotic Lymphocytes of H. Sapiens lose nucleosomes in GC-rich promoters. Plos Computational Biology. 10(7), e1003760.

[13] Hughes, A. L. and Rando, O. J. (2014) Mechanisms underlying nucleosome positioning in vivo. Annu Rev Biophys., 43, 41-63.

[14] Ioshikhes, I. et al. (2011) Variety of genomic DNA patterns for nucleosome positioning. Genome Res., 21(11), 1863-1871.

[15] Iyer,V.R. (2012) Nucleosome positioning: bringing order to the eukaryotic genome. Trends Cell Biol., 22(5), 250-256.

[16] Jiang, M. et al. (2008) uShuffle: A useful tool for shuffling biological sequences while preserving the k-let counts. BMC Bioinformatics, 9,192.

[17] Li, H. and Durbin, R. (2010). Fast and accurate long-read alignment with Burrows Wheeler transform. Bioinformatics,26(5), 589–595.

[18] Radman-Livaja, M. and Rando, O. (2010) Nucleosome positioning: how is it established, and why does it matter? Dev Biol. 339(2), 258-266.

[19] Lowary, P. T. and Widom, J. (1998) New DNA sequence rules for high affinity binding to histone octamer and sequence-directed nucleosome positioning. J. Mol. Biol., 276(1), 19-42.

[20] Luger, K. et al. (1997) Crystal structure of the nucleosome core particle at 2.8A resolution. Nature, 389(6648), 251-260.

[21] Nellore, A. et al. (2012) NSeq: a multithreaded Java application for finding positioned nucleosomes from sequencing data. Front Genet., 3, 320.

[22] Polishko, A. et al. (2012) NOrMAL: accurate nucleosome positioning using a modified Gaussian mixture model. Bioinformatics, 28(12), i242-i249.

[23] Quinlan,A.R.(2014). BEDTools: The Swiss-Army Tool for Genome Feature Analysis. Curr Protoc Bioinformatics,47:1–34.

[24] Quintales, L. et al. (2015) Comparative analysis of methods for genome-wide nucleosome cartography. Brief. Bioinformatics, 4, 576-587.

[25] Schones, D.E. et al. (2008) Dynamic regulation of nucleosome positioning in the human genome. Cell, 132(5), 887-898.

[26] Scipioni, A. and De Santis, P. (2011) Predicting nucleosome positioning in genomes: physical and bioinformatic approaches. Biophys. Chem., 155(2-3), 53-64.

[27] Segal, M. R. (2008) Re-cracking the nucleosome positioning code. Stat Appl Genet Mol Biol., 7(1), Article14.

[28] Snyder, M. et al. (2016) Cell-free DNA comprises an in vivo nucleosome footprint that informs its tissues-of-origin. Cell, 164(1-2), 57-68.

[29] Struhl, K. and Segal, E. (2013) Determinants of nucleosome positioning. Nat. Struct. Mol. Biol., 20(3), 267-273.

[30] Sun, H. Et al. (2015) ACF chromatin-remodeling complex mediates stress-induced depressive-like behavior. Nat. Med., 21(10), 1146-1153.

[31] Teif, V. B. (2016) Nucleosome positioning: resources and tools online. Brief. Bioinformatics, 17(5), 745-757.

[32] Tirosh, I. (2012) Computational analysis of nucleosome positioning. Methods Mol. Biol., 833, 443-449.

[33] Yang , D. and Ioshikhes, I. (2017) Drosophila H2A and H2A.Z nucleosome sequences reveal different nucleosome positioning sequence patterns. J Comput Biol. 24(4), 289-298.

[34] Zhang, Y. et al. (2008) Identifying positioned nucleosomes with epigenetic marks in human from ChIP-Seq. BMC Genomics, 9, 537

[35] Zhang, J. et al. (2018) LeNup: learning nucleosome positioning from DNA sequences with improved convolutional neural networks. Bioinformatics, 30(10), 1705-1712.

[36] Zhou, X. et al. (2016) A computational approach to map nucleosome positions and alternative chromatin states with base pair resolution. eLife, 5.
