# Supplementary material for: Nucleosome positioning sequence patterns as packing or regulatory
Source: PLoS Comput Biol. 2020 Jan 27;16(1):e1007365. doi: 10.1371/journal.pcbi.1007365 (PMC7004410; doi:10.1371/journal.pcbi.1007365)
Supplement: S2 Appendix — This Appendix presents multiple plots of patterns in nucleosomal DNA of mouse and human with additional analysis and details on distributions of peaks. It contains a short summary on importance of superhelical locations. (PDF) [file pcbi.1007365.s002.pdf]

Comparative analysis of nucleosome patterns in mouse and human cells and importance of superhelical locations. Supplementary Appendix S2 to ‘Patterns in nucleosomal DNA as packing or regulatory‘

Contents

1 Dinucleotide frequency profiles across various conditions **2**
1.1 Dinucleotide patterns in human and mouse 2
1.2 Comparison of centered paterns of dinucleotide frequency 3

2 Synchronicity of peaks and positional preferences **5**
2.1 Peak alignment in nucleosomal DNA patterns across various conditions 5
2.2 Positional preferences of dinucleotides 6

3 Importance of Superhelical Locations **8**
3.1 Nucleosome stability 8
3.2 Histone-DNA interactions 8
3.3 Remodeling and post-translational modifications 9

1 Dinucleotide frequency profiles across various conditions

1.1 Dinucleotide patterns in human and mouse

Similarities between dinucleotide profiles across conditions in human and mouse are prominent in WW (Figure 1a) and SS (Figure 1b) patterns in corresponding peaks and valleys. Consistently, the peaks in the WW profiles are in phase for human CD4+ and mouse cells. Most of the WW versus SS peaks and most of the RR versus YY peaks are off phase in both human and mouse cells.

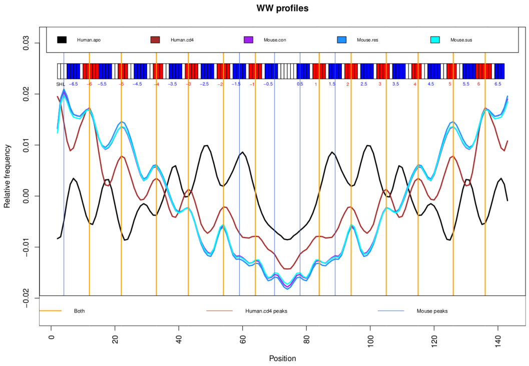

(a) WW profiles

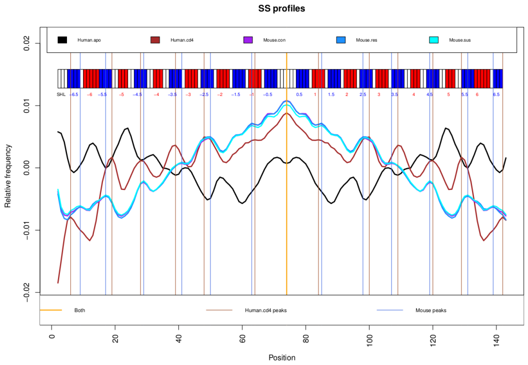

(b) SS profiles

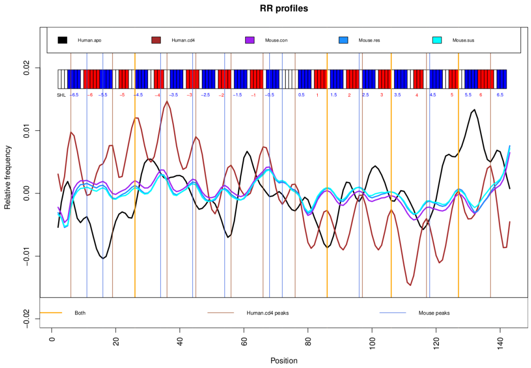

(c) RR profiles

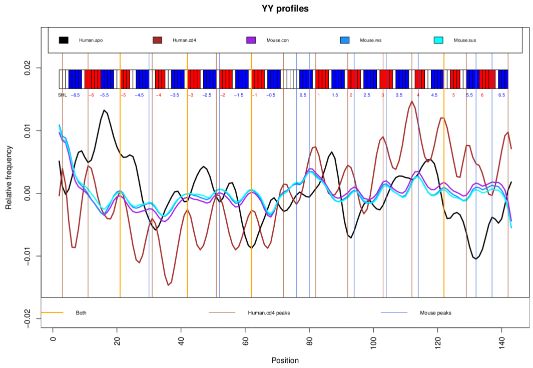

(d) YY profiles

Figure 1: Superimposed centered dinucleotide frequency patterns in all conditions. Peaks coinciding in both organisms shown by orange lines, peaks specific to mouse shown by blue lines and peaks in human are shown by brown lines. The rule at the top represents superhelical locations SHL along nucleosomal DNA: red segments shows major groves and blue segments-minor.

1.2 Comparison of centered paterns of dinucleotide frequency

Figures 2, 3,4,5 show centered dinucleotide frequency patterns in nucleosomal DNA sequences. In each Figure the arrangement is following: human CD4+ (`top left`) and apoptotic lymphocyte (`top right`) cells and nucleus accumbens cells (NAC) of mouse brain in control (`bottom left`) and stress susceptible (`bottom right`) mice. While peaks in the nucleosome patterns of human CD4+ and mouse cells are in phase, the corresponding peaks and valleys of the patterns in apoptotic lymphocytes are off-phase.

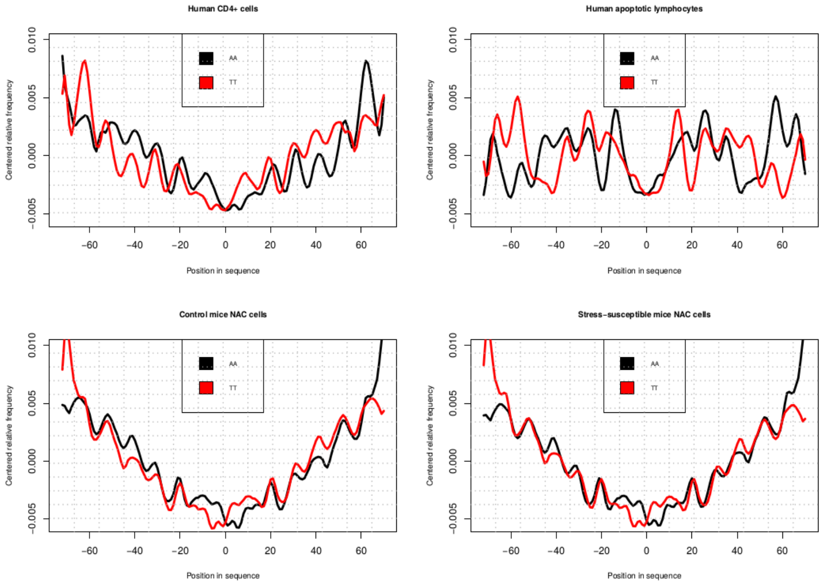

Figure 2: AA - black and TT - red.

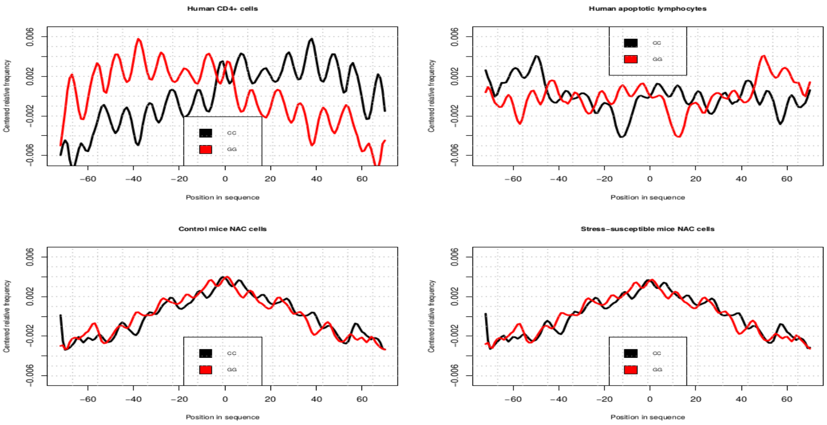

Figure 3: CC - black and GG - red.

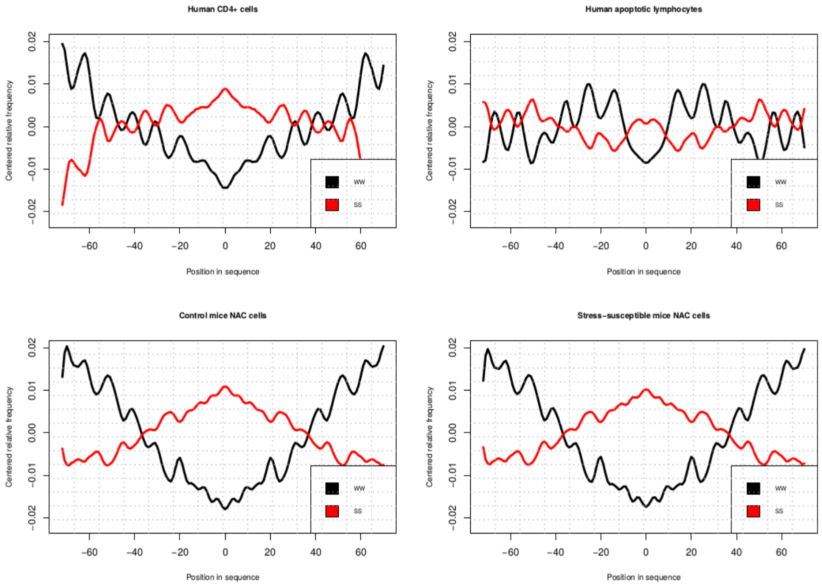

Figure 4: WW - black and SS - red.

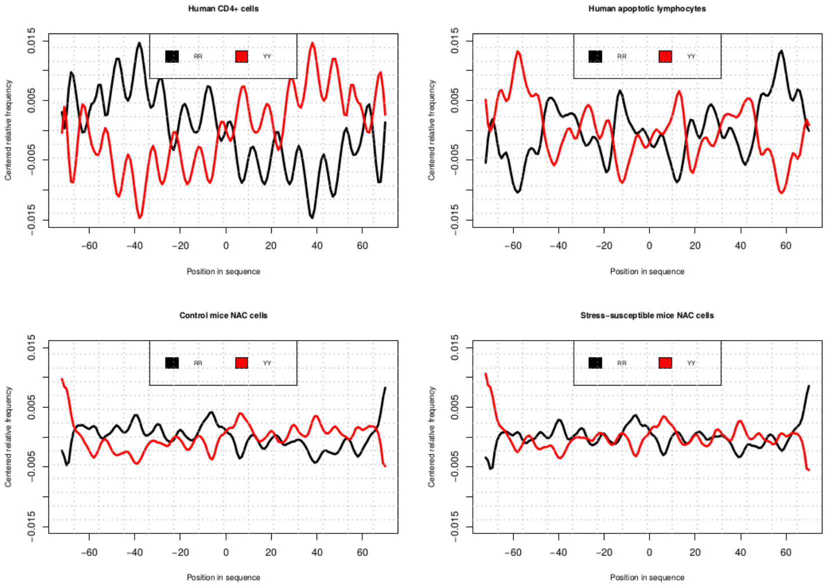

Figure 5: RR - black and YY - red.

2 Synchronicity of peaks and positional preferences

2.1 Peak alignment in nucleosomal DNA patterns across various conditions

Dinucleotide peaks in nucleosomal DNA appear in specific locations that are synchronous in human and mouse organism and in different conditions as illustrated in Figure 6. The peaks in general tend to co-occur at certain distances. Frequency distribution of distances between the co-occurring peaks showed that peaks mostly co-occur at intervals separated by a step of 10 +/- 1 base pairs.

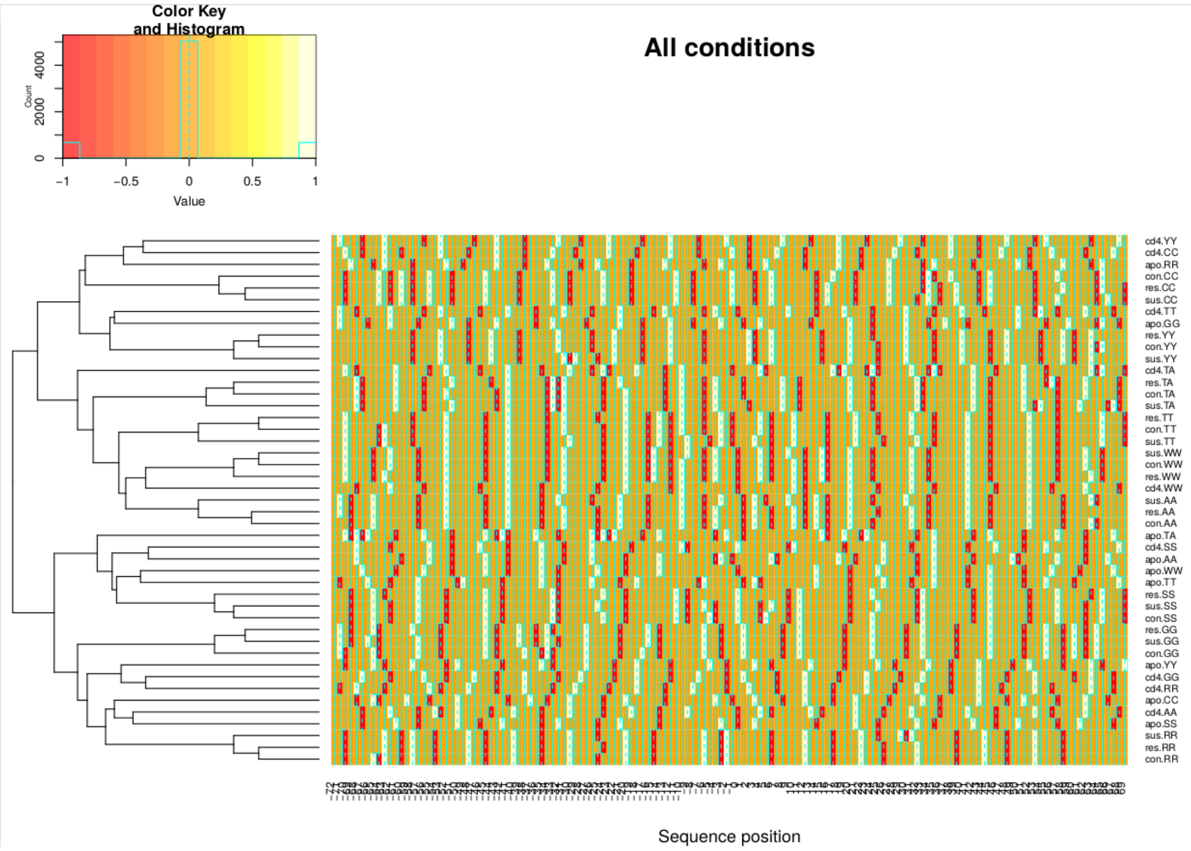

Figure 6: Indicator sequences for AA, TT, AT, CC, GG, WW, SS, RR and YY dinucleotide patterns are shown in all conditions as a heatmap. A dendrogram on the left represents patterns grouped by Euclidean distance and clearly represents coinciding maxima and minima across conditions. Dinucleotide frequency patterns in nucleosomal DNA are centered on a dyad. The labels on the right cd4 and apo corresponds to human CD4+ and apoptotic lymphocyte cells; the con, sus and res correspond to mouse NAC in control, susceptible and resilient to stress conditions.

2.2 Positional preferences of dinucleotides

A frequency of peaks simultaneously occurring in each position in all conditions is shown in Figure 7. For some pairs of the dinucleotides their maximums occurr together only in human or only in mouse. Figure 8 shows an order of positions that were used more than eight times by different dinucleotide classes. The hub positions occurr at $\pm 10, 11$ base pair steps around the dyad. Positional preferences elucidated in this study are summarized in Table 1. The hub positions are used by a majority of dinucleotides, but some positions such as -45, -31, -20 20, 31, 45 are simultaneously used by incompatible dinucleotides.

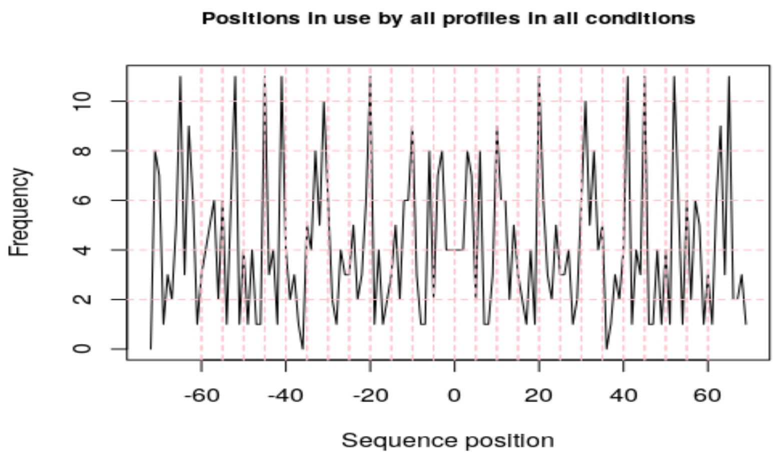

Figure 7: Positions in use by all profiles in all conditions

Table 1: Positions, identities and conditions of dinucleotides of high requency of occurrence.

Position	Majority	Conditions of majority	Minority	Conditions of minority
-52	AA,TT,WW	CD4+, Mouse all	RR	Apoptotic
-45	CC,GG,SS	Mouse all	AA,WW	Apoptotic
-41	AA,TT,WW	CD4+, Mouse all	-	-
-31	AA,TT,WW	CD4+, Mouse all	YY	Mouse stress susceptible
-20	TT,WW, RR	CD4+, Mouse all	CC	Apoptotic
-11	CC,SS	Mouse all	-	-
-10	AA,TT,WW	CD4+, Mouse all	SS	CD4+
10	AA,TT,WW	CD4+, Mouse all	SS	CD4+
11	GG, SS	Mouse all	-	-
20	AA,WW,YY	CD4+, Mouse all	GG	Apoptotic
31	AA,TT,WW	CD4+, Mouse all	RR	Mouse stress susceptible
41	AA,TT,WW	CD4+, Mouse all	-	-
45	CC,GG,SS	Mouse all	TT,WW	Apoptotic
52	AA,TT,WW	CD4+, Mouse all	YY	Apoptotic

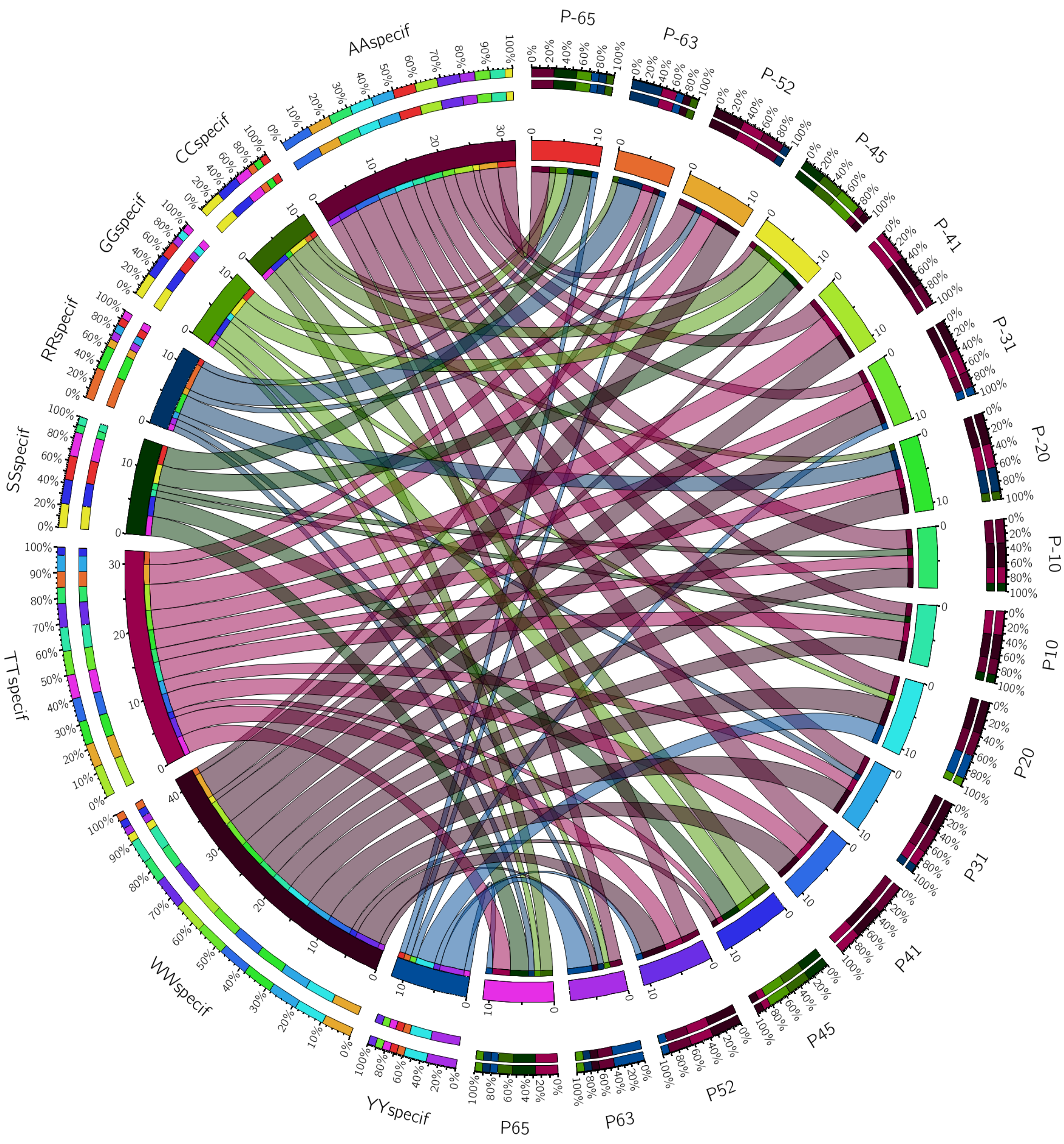

Figure 8: Hub positions preferred by different classes of dinucleotides visualized as a circos plot. Compatible dinucleotides are color coded: AA, TT and WW have purple shades; CC, GG and SS have green shades; and RR, YY have blue shades

3 Importance of Superhelical Locations

Statistically defined relations between nucleosome positioning and covalent chromatin modification were noted – but it is not clear yet how specific composition of DNA sequence might interact with these processes and affect nucleosome positioning and remodelling. In the following we briefly summarized current knowledge about importance of specific SHL zones trying to provide more context to the regularities observed in this work.

In this study we observed multiple peaks from all organisms in SHL zones ± 2 , ± 3 and ± 4. The SHL ± 2, ± 3 are zones in which chromatin remodelers interact with DNA and in SHL ± 4 zone histone H2A tail interacts with DNA which makes it a less stable zone. Peak distributions across SHLs were different in mouse compared to human in SHL zones $\pm 1.5, 4$ and ± 4.5 which also play roles in nucleosome dynamics. Most of DNA deformations take place in the SHL± 1.5 zone. It has an increased binding of DNA proteins and it is most susceptible to DNA damage.

3.1 Nucleosome stability

The H3-H4 tetramer is the most stable part of the formed nucleosome. The H2A and H2B are more mobile and H1 is very dynamic. Main histone tails occur in minor grooves [3]. The nucleosomes can adopt multiple conformations providing a structural basis for nucleosome plasticity which in turn may contribute to chromatin regulation in many processes [4]. The SHL $\pm 1.5, 2.5$ and SHL $\pm 6, 7$ are the most active zones in which DNA shows the largest movements between the structures and H3 alpha helices show the largest rearrangements. Nucleosomes wrap and unwrap spontaneously interchanging between a 250ms in a compacted state 10-50 ms in an unwrapped state where the unwrapping occurs from the ends of the nucleosome [5]. The out-of-planar bending (by SHLs ± 3.5 and ± 4.5) and in-planar rolling (by SHLs ± 6 and ± 6.5) motions of the super helical DNA in general are responsible for the breathing motion – wrapping and unwrapping of nucleosome [6].

3.2 Histone-DNA interactions

The greatest numbers of interactions between histone core and DNA are located at the central bound DNA region, SHLs ± 0.5 at the dyad axis. The least number of interactions are found at the entry-exit regions in SHLs ± 5.5 and ± 6.5. Other SHLs have an intermediate number of direct hydrogen bonds [7]. Pressure points, are very important for nucleosome stability [8]. The SHL ± 2.5 zone has increased unwrapping because of the acetylation of H4 residues and because histone H2B N-terminal extension makes numerous contacts with DNA at that site. Both, H2A/H2B and H3/H4 contact the DNA via their $\alpha 1 - \alpha 1$ helices and two L1-L2 loops, with histone H2A/H2B binding to superhelical locations ± 3 to ± 6. The histone H2A tail exits adjacent to the minor groove at SHL ± 4–4.5 and residues in this helix contact the DNA. Histone H2B N-terminal extension makes numerous contacts with both DNA coils at SHL $\pm 4.5 - 5$ and SHL $\pm 2.5 - 3$ [9]. The DNA deformations predominantly occur around the sites SHL ± 1.5 and ± 2 (in the central part of nucleosomal DNA interacting with the H3/H4 tetramer), and at the sites SHL ± 4.5 (interacting with the H2B histones) [1]. The sites at SHL ± 1.5 and ± 4.5 are sites where the helix is stretched and it is more reactive to DNA damaging agents [10]. The DNA superhelical location (SHL) ± 1.5 is a known hot spot for

DNA binding/damaging molecules. Energetically significant interaction stable sites are ±3 and less stable sites are ±4 and ±5 where H2A/H2B bind to the DNA.

3.3 Remodeling and post-translational modifications

In addition to DNA sequence other mechanisms modulate nucleosome stability and dynamics. One involves chemically altering the histones themselves and greatly increases the DNA accessibility. These changes can be post-translational modifications (PTMs) the best-studied of which are acetylation, methylation, phosphorylation, ubiquitynation, and ADP-ribosylation of which acetylation of histone tails destabilize nucleosomes. Increased DNA unwrapping from the nucleosome happens with acetylation of two residues, H4(K77) and K4(K79) near superhelical location (SHL) ±2.5. A second mechanism influencing nucleosome stability and dynamics is through remodelling factors that must transiently disrupt DNA protein contacts [2]. Remodelers are grouped into INO80, SWI/SNF, CHD and ISWI families that collectively shape the nucleosome landscape [11]. INO80 binds into SHL -6 and disrupts H2A/H2B DNA contacts. Arp5-Ies6 binds about 7-8 bases at SHL-2/-3. The ISWI and SWI/SNF remodelling enzymes engage nucleosomal DNA at SHL +/- 2 [12] at which a detachment of DNA could be achieved inside the nucleosomal structure near the dyad axis. Regions with active H3K4 H3K9 marks are characterized by lower nucleosome density, while inactive chromatin marks such as methylation H3K9 H3K27 are characterized by higher nucleosome density [13].

All these observations taken together show that all SHLs play important role in processes regulating nucleosome stability. A better understanding of how specific dinucleotides at certain SHL may affect these processes is a subject of current and future research.

References

[1] Cui, F. and Zhurkin, V. (2010) Structure-based Analysis of DNA Sequence Patterns Guiding Nucleosome Positioning in vitro. J Biomol Struct Dyn. 27(6):821-841.

[2] Bowman, G.D. and Poirier, M.G. (2015) Post-translational modifications of histones that influence nucleosome dynamics. Chem Rev 115(6): 2274-2295.

[3] Davey, C.A., Sargent, D.F., Luger, K., Maeder, A.W. and Richmond, T.J. (2002) Solvent mediated interactions in the structure of the nucleosome core particle at 1.9 a resolution. J Mol Biol 319(5): 1097-113.

[4] Bilokapic, S., Strauss, M. and Halic, M. (2018) Structural rearrangements of the histone octamer translocate DNA. Nat commun 9(1): 1330 doi: 10.1038/s41467-018-03677-z.

[5] Rippe, K., Mazurkiewicz, J. and Kepper, N. (2008) Interactions of histones with DNA: nucleosome assembly, stability, dynamics, and higher order structure. In Dias, R. and Lindman, B. (ed.) DNA Interactions with Polymers and Surfactants. John Wiley & Sons, Inc. Hoboken NJ USA. pp. 135-172.

[6] Rajagopalan, M., Balasubramanian, S., Ioshikhes, I. and Ramaswamy, A. (2017) Structural dynamics of nucleosome mediated by acetylations at H3K56 and H3K115, 122. Eur Biophys J 46(5): 471-484.

[7] Nikova, D.N., Pope, L.H., Bennink, M.L., van Leijenhorst-Groener, K.A., van der Werf, K. and Greve, J. (2004) Unexpected binding motifs for subnucleosomal particles revealed by atomic force microscopy. Biophys J 87(6):4135-45.

[8] McGinty, R.K. and Tan, S. (2015) Nucleosome structure and function. Chem. Rev 115(6):2255-2273.

[9] Wyrick, J.J., Kyriss, M.N. and Davis, W.B. (2012) Ascending the nucleosome face: recognition and function of structured domains in the histone H2A–H2B dimer. Biochim Biophys Acta 1819(8): 892-901.

[10] Taylor, J.S. (2015) Design, synthesis, and characterization of nucleosomes containing site-specific DNA damage. DNA repair 36: 59-67.

[11] Eustermann, S., Schall, K., Kostrewa, D., Lakomek, K., Strauss, M., Moldt, M. and Hopfner, K.P. (2018) Structural basis for ATP-dependent chromatin remodelling by the INO80 complex. Nature 556(7701): 386-390.

[12] Narlikar, G.J., Sundaramoorthy, R. and Owen-Hughes, T. (2013) Mechanisms and functions of ATP-dependent chromatin-remodeling enzymes. Cell 154(3): 490-503.

[13] Collings, C.K. and Anderson, J.N. (2017) Links between DNA methylation and nucleosome occupancy in the human genome. Epigenetics chromatin 10(1): 18 doi: 10.1186/s13072-017-0125-5.
