# Supplementary material for: Nucleosome positioning sequence patterns as packing or regulatory
Source: PLoS Comput Biol. 2020 Jan 27;16(1):e1007365. doi: 10.1371/journal.pcbi.1007365 (PMC7004410; doi:10.1371/journal.pcbi.1007365)
Supplement: S3 Appendix — This appendix shows ECDFs of maximal Person CC obtained in a computational mapping of nucleosome sequences by the dinucleotide distribution patterns analyzed in this study. The computational nucleosome mapping was done in sequences from promoters and gene body loci (mm9 genome build) in which regulatory events of nucleosome occupancy change took place. The ECDFs are also shown for Pearson CC in random +1 nucleosome sequences. Computational nucleosome mapping data is presented for stress-susceptible (sus) and stress-resilient (res) mice sequences. (PDF) [file pcbi.1007365.s003.pdf]

Supplementary S3 Appendix.

Empirical cumulative distribution functions (ECFD) of maximal Pearson correlation coefficients (CC) for each pattern in sequences from promoters and gene body loci (mm9 genome build) in which regulatory events of nucleosome occupancy change took place in stress-susceptible (sus) and stress-resilient (res) mice. ECFDs are also shown for Pearson CC in randomly selected +1 nucleosome sequences in stress-susceptible (sus) and stress-resilient (res) mice.

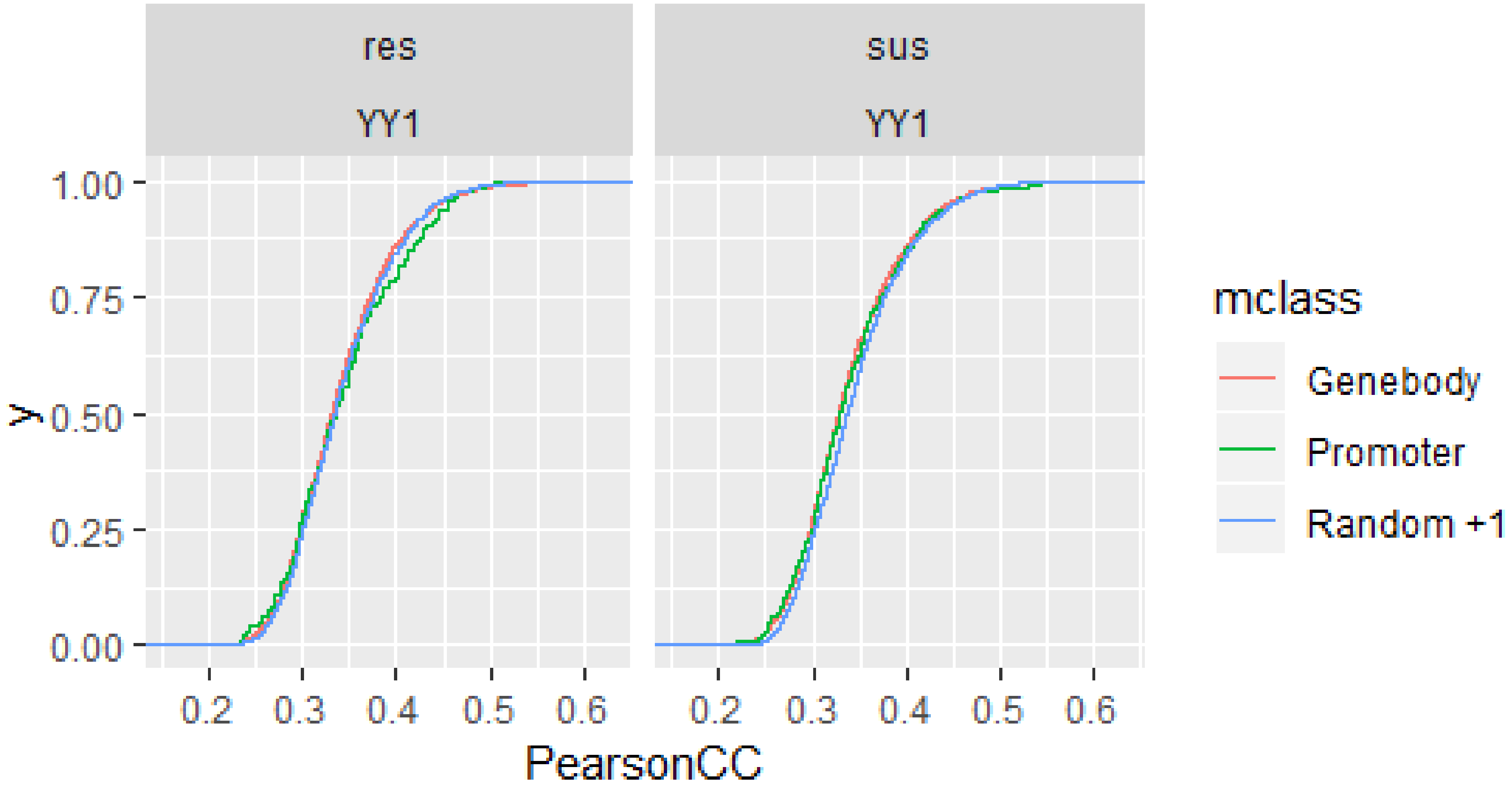
res
YY1
sus
YY1
1.00
0.75
0.50
0.25
0.00
y
0.2
0.3
0.4
0.5
0.6
PearsonCC
mclass
Genebody
Promoter
Random +1

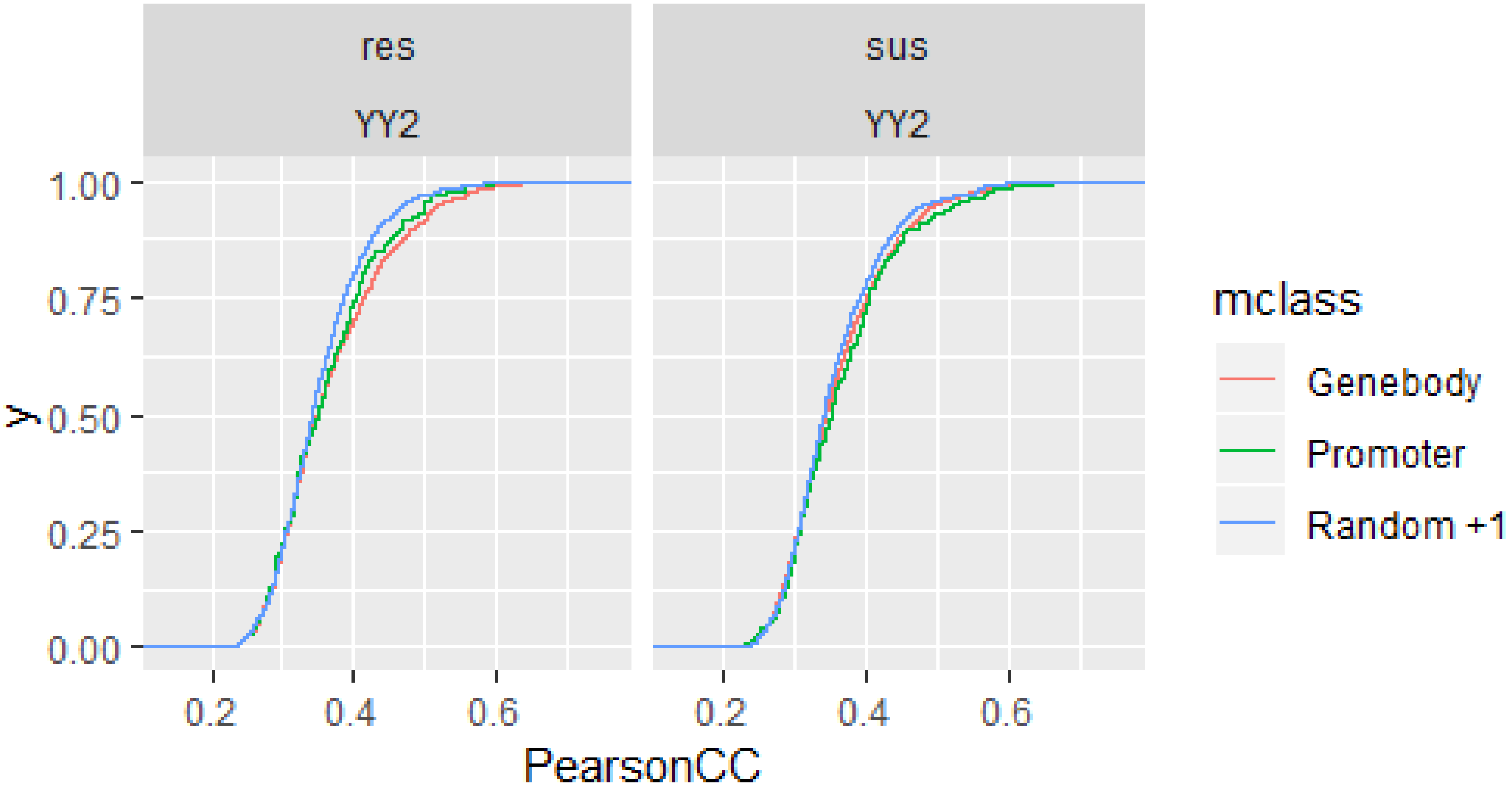

res
YY2
sus
YY2
1.00
0.75
0.50
0.25
0.00
y
0.2
0.4
0.6
PearsonCC
mclass
Genebody
Promoter
Random +1

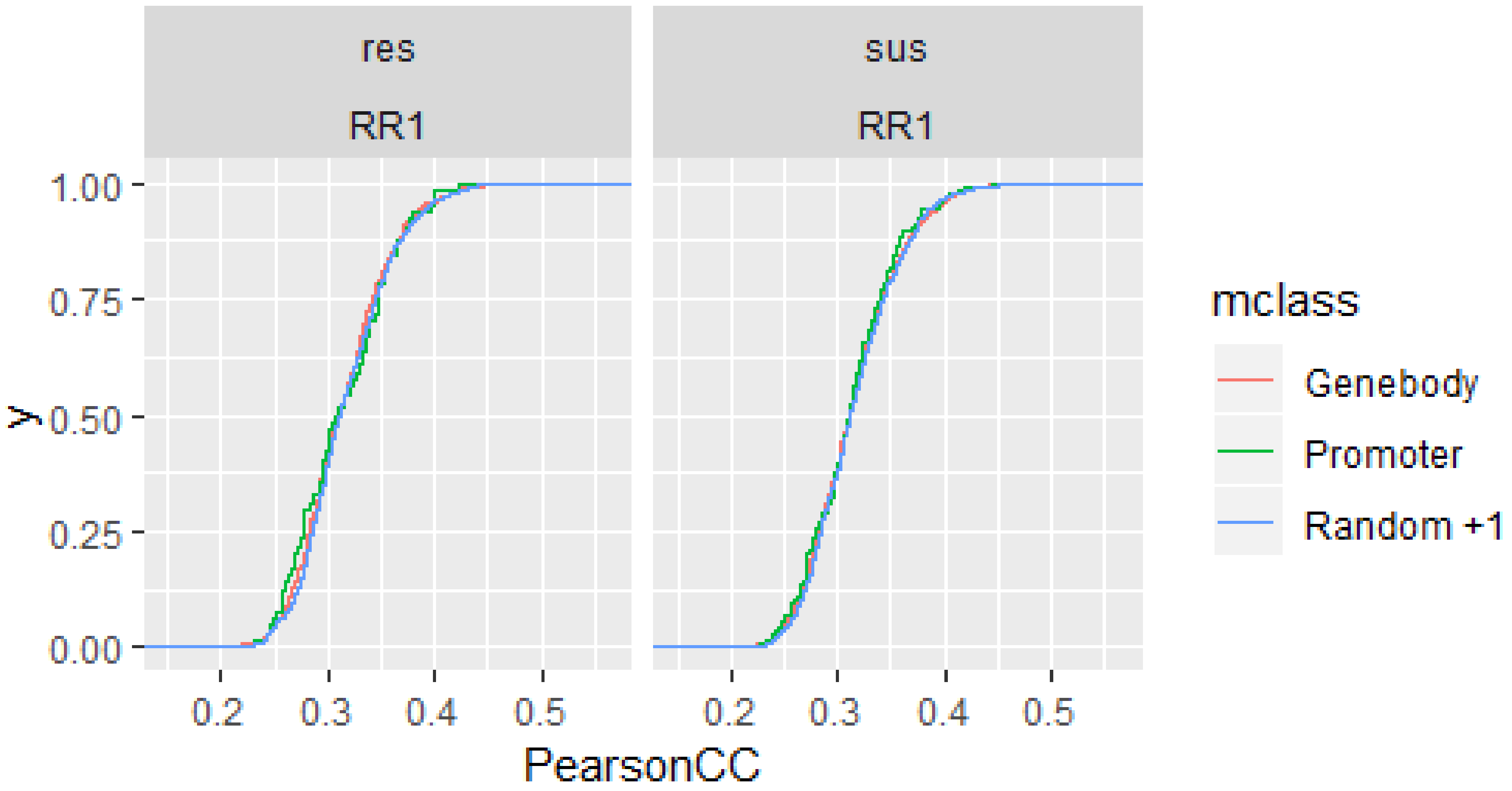
res
RR1
sus
RR1
1.00
0.75
0.50
0.25
0.00
y
0.2
0.3
0.4
0.5
PearsonCC
mclass
Genebody
Promoter
Random +1

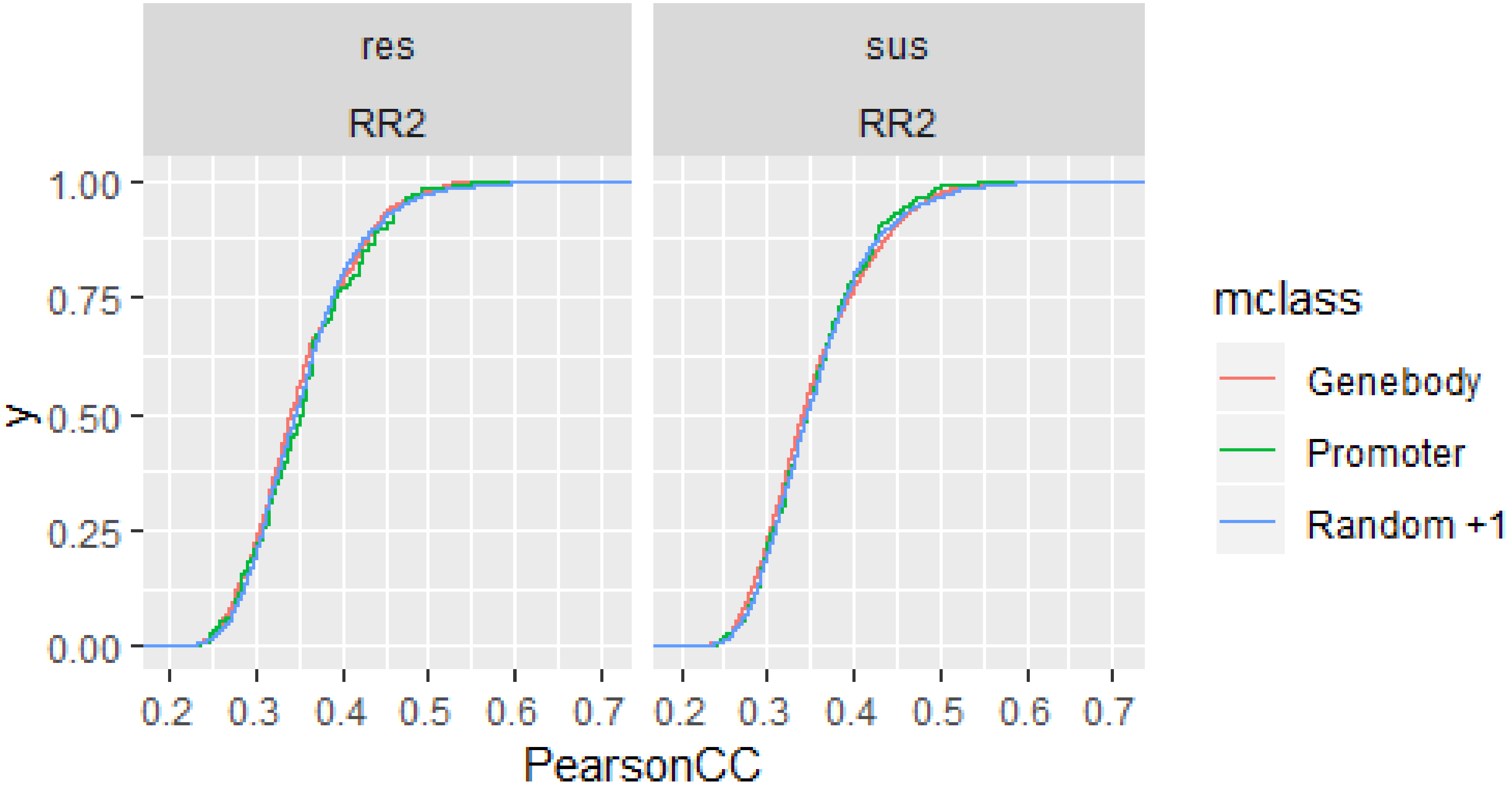

res
RR2
sus
RR2
y
1.00
0.75
0.50
0.25
0.00
0.2
0.3
0.4
0.5
0.6
0.7
PearsonCC
mclass
Genebody
Promoter
Random +1

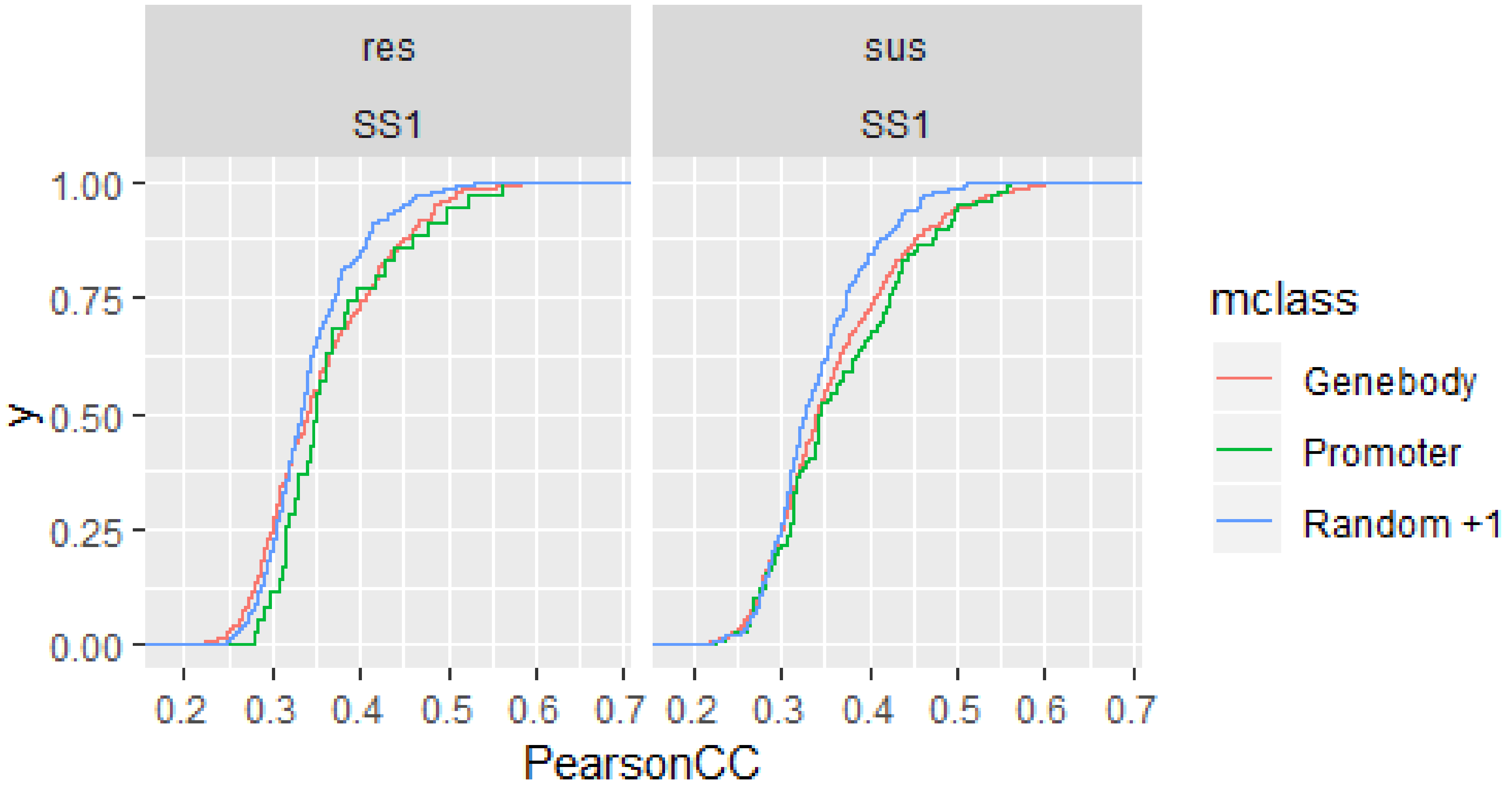

res
SS1
sus
SS1
1.00
0.75
0.50
0.25
0.00
y
0.2
0.3
0.4
0.5
0.6
0.7
PearsonCC
mclass
Genebody
Promoter
Random +1

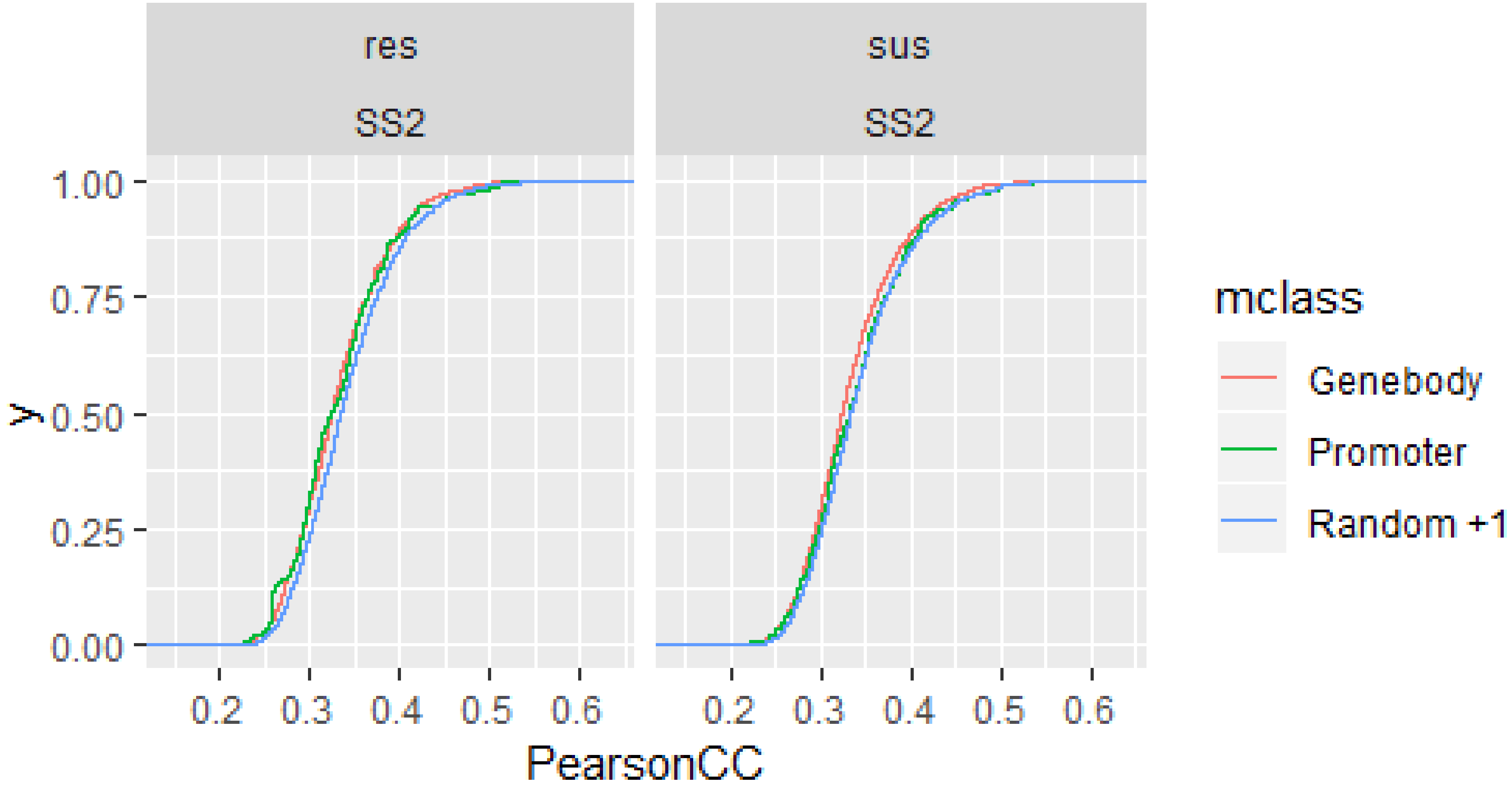

res
SS2
sus
SS2
1.00
0.75
0.50
0.25
0.00
y
0.2
0.3
0.4
0.5
0.6
PearsonCC
mclass
Genebody
Promoter
Random +1

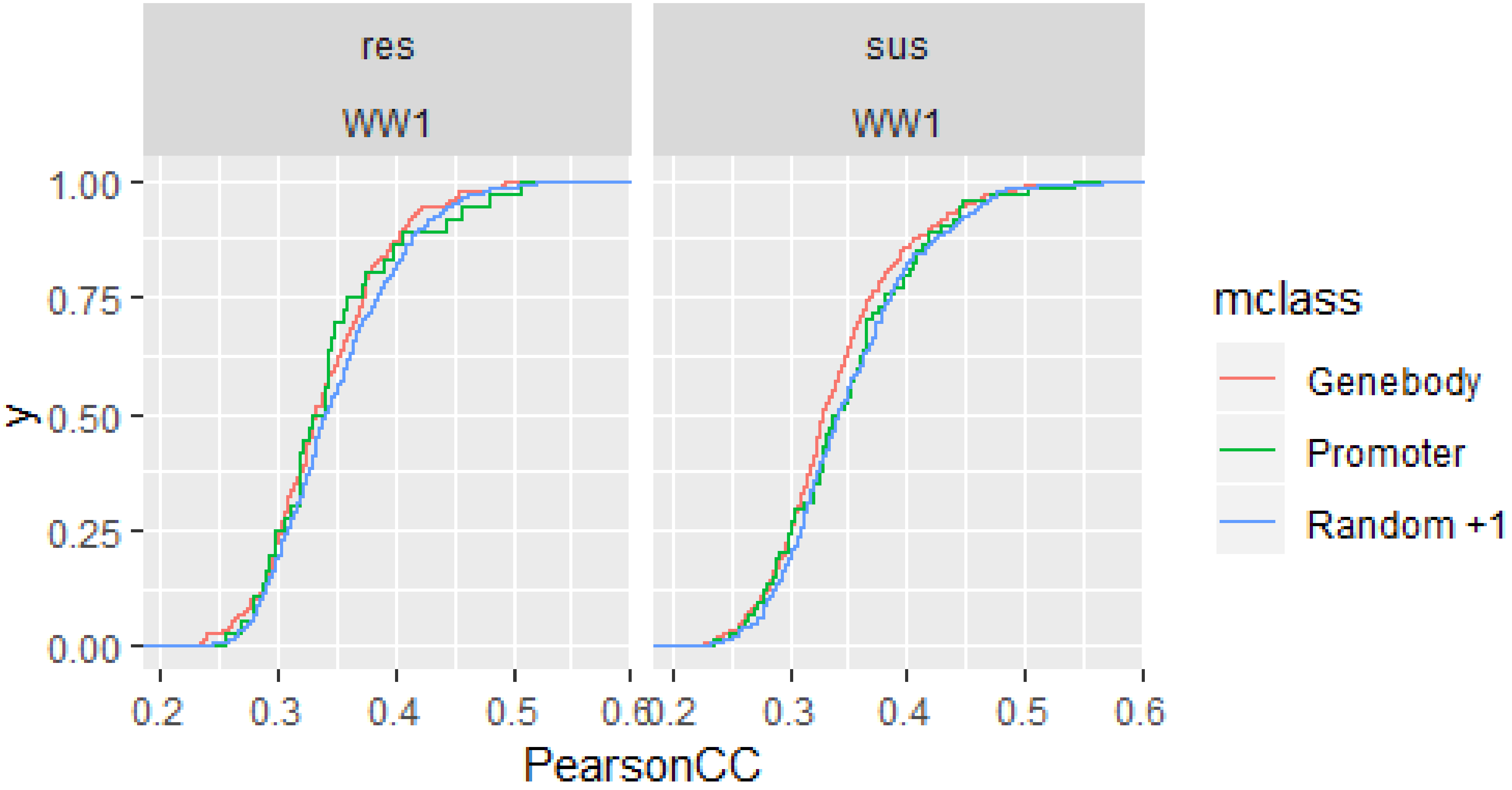

res
WW1
sus
WW1
1.00
0.75
0.50
0.25
0.00
y
0.2
0.3
0.4
0.5
0.6
PearsonCC
mclass
Genebody
Promoter
Random +1

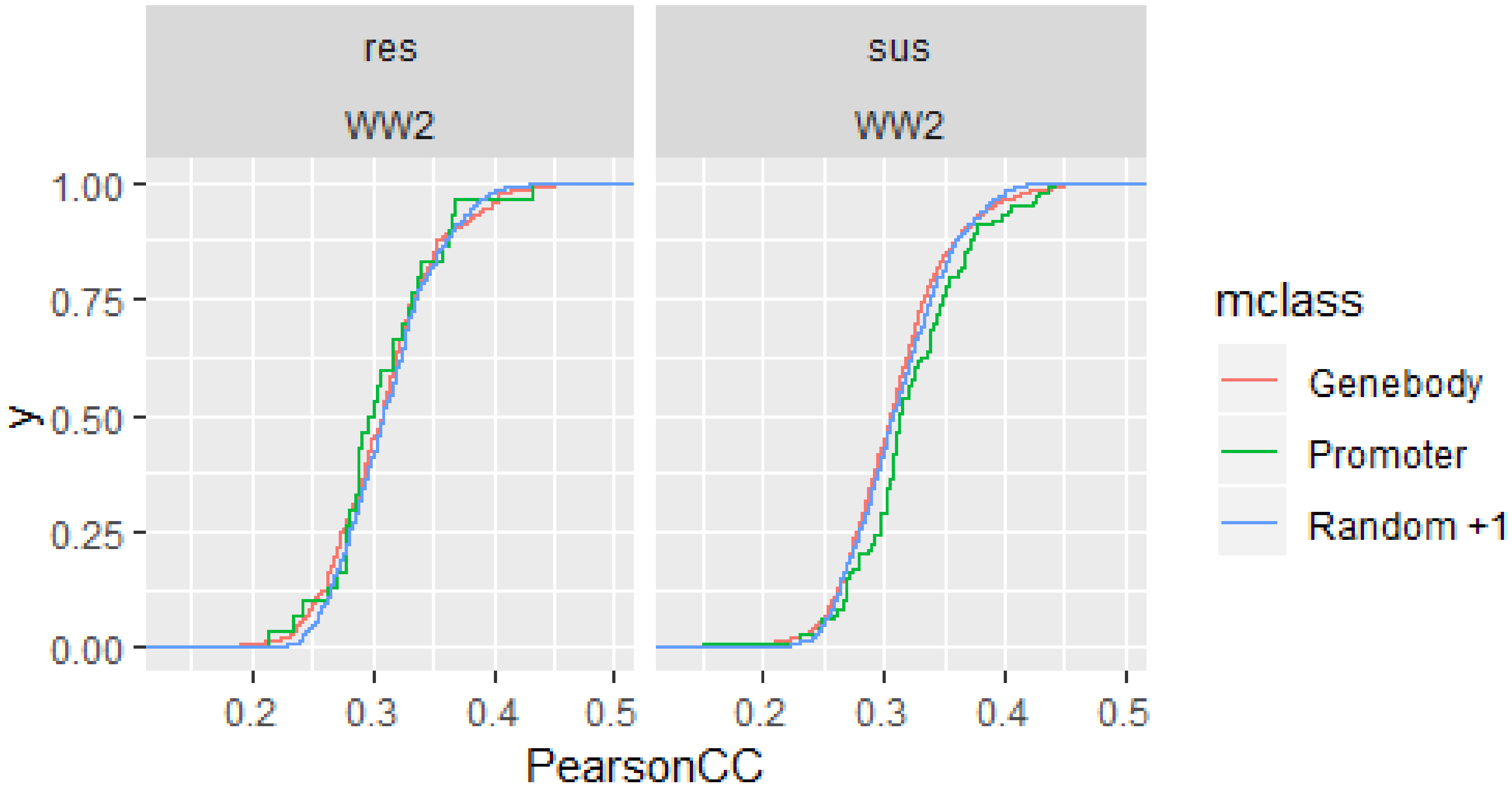
res
WW2
sus
WW2
1.00
0.75
0.50
0.25
0.00
y
0.2
0.3
0.4
0.5
PearsonCC
mclass
Genebody
Promoter
Random +1
